# Atomically thin gold embedded in inkjet-printed PVA hydrogels: flexible catalysts for ambient phenol degradation

**DOI:** 10.1039/d5na00968e

**Published:** 2026-01-12

**Authors:** Nizzy James, Sean Collins, Quentin Ramasse, Kevin Critchley, Stephen D. Evans

**Affiliations:** a School of Physics and Astronomy, Bragg Centre for Materials Research, University of Leeds Woodhouse Lane LS2 9JT Leeds UK s.d.evans@leeds.ac.uk; b School of Physics and Astronomy and School of Chemical and Process Engineering, University of Leeds Woodhouse Lane LS2 9JT Leeds UK; c SuperSTEM Laboratory, SciTech Daresbury Campus Keckwick Lane WA4 4AD Daresbury UK; d Bragg Centre for Materials Research, School of Chemical and Process Engineering and School of Chemistry, University of Leeds Woodhouse Lane LS2 9JT Leeds UK

## Abstract

Inkjet-printed gold nanotape (AuNTp) structures embedded in polyvinyl alcohol (PVA) hydrogels provide a reusable, high-surface-area platform for catalytic degradation of phenol and 4-nitrophenol (4-NP) under ambient conditions. AuNTps, featuring distinct three-dimensional “heads” and atomically thin quasi-one-dimensional “tails”, enhanced catalytic activity in both reduction and oxidation reactions. Compared to spherical gold nanoparticles (AuNPs), AuNTps are nearly twice as catalytically efficient for 4-NP reduction on a per-mass basis, reflecting the influence of anisotropic morphology on surface-sensitive electron transfer. In contrast, phenol oxidation shows weaker morphology dependence, likely proceeding through hydroxyl radical-mediated pathways that are less sensitive to catalyst shape or facet structure. To enable rapid substrate diffusion and facilitate reuse, AuNTps were formulated into PVA inks and inkjet-printed into micrometre-thick hydrogel mesh architectures (8 to 15 µm thick). Although printed meshes show reduced activity relative to free AuNTps in solution, they achieve a nearly fourfold increase in mass-normalised rate constants for 4-NP reduction compared to drop-cast gels (0.24 × 10^4^*vs.* 0.07 × 10^4^ min^−1^ g^−1^) and achieve 26% phenol, a common water pollutant, in 4 hours at room temperature, with consistent performance over multiple cycles. These findings demonstrate the potential of inkjet-printed nanozyme hydrogels as scalable, heterogeneous catalysts. Further improvements may be achieved by optimising catalyst-matrix interactions to reduce diffusion and accessibility barriers. This work addresses a significant challenge in nanozyme catalysis: translating high-performance nanomaterials into practical, reusable formats suitable for environmental remediation.

## Abstract 2

Printable PVA hydrogels embedded with gold nanotapes enable reusable, high-efficiency catalysts for pollutant degradation at room temperature. Inkjet-printed meshes boost diffusion, achieving 4× faster 4-NP reduction than bulk gels.

## Graphical abstract

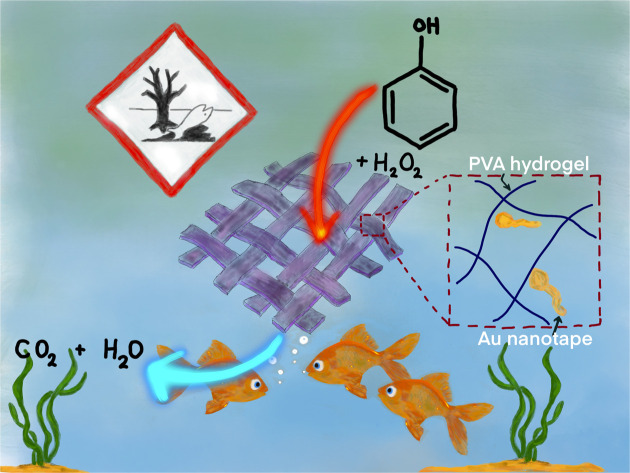

## Introduction

Two-dimensional (2D) nanomaterials, such as graphene, have garnered attention due to their distinctive electronic, mechanical, and surface properties resulting from their reduced dimensionality.^1,2^ Ultrathin metal nanostructures represent a subclass of 2D materials with potential applications in bioimaging, therapy, sensing, and catalysis. In particular, 2D noble metal nanomaterials are of interest because their thin structure provides a high surface area-to-volume ratio and a large number of exposed active sites, facilitating improved catalytic interactions.^3,4^

Recently, we reported a novel synthesis method for producing freestanding, atomically thin gold nanosheets (AuNS) in aqueous media.^5^ These AuNS demonstrated a tenfold increase in catalytic efficiency for reducing 4-nitrophenol (4-NP) compared to spherical AuNPs. Furthermore, we have demonstrated that modifying the synthesis route yields various gold nanostructures, including quasi-1D nano-tapes (AuNTps) and nano-pinecones.^6^ The AuNTps are of particular interest as they exhibit the highest catalytic efficiency, suggesting that edge sites play a crucial role in determining the catalytic activity of these materials.^7^ In addition to their enhanced catalytic properties, AuNSs and AuNTps also exhibited peroxidase-mimicking activity, verified by the oxidation of 3,3′,5,5′-tetramethylbenzidine (TMB) and 3,3′-diaminobenzidine (DAB).^7^ However, the use of such nanomaterials as homogeneous catalysts or enzyme mimics is often limited by the need for their separation and recovery after each use, which can be a non-trivial process. Embedding nanomaterials into macroscale hydrogels provides a practical alternative, preserving catalytic activity while enabling heterogeneous catalysis. In this form, reactants can diffuse through the porous hydrogel and interact with immobilised nanoparticles within the polymer matrix.^8–10^ This approach has been extended beyond conventional laboratory settings, including applications in wound dressings and injectable formulations, where wound fluid can interact with the hydrogel to facilitate nanozyme-mediated reduction of reactive oxygen species, thereby supporting tissue repair.^2,7,11^ Furthermore, these materials have potential applications in portable sensing platforms, such as nanozyme-driven colourimetric assays for the visual detection of environmental analytes.^5–7^

Poly (vinyl alcohol) (PVA) based hydrogels are flexible, biocompatible, and inexpensive, exhibiting high water absorbency (>90%)^12^ and tuneable mechanical properties. Additionally, PVA gels can be easily processed into various morphologies, including tiles, films, and membranes, and have been widely utilised in wastewater treatment applications.^13–15^ Previously, we have demonstrated the incorporation of AuNPs into PVA hydrogel tiles, showing their capacity to act as scaffolds for supported particles.^7^ However, these bulk hydrogel studies revealed significantly reduced reaction times due to the slow diffusion of the reagents into the hydrogel structures. Inspite of the reduced diffusion within hydrogels the hydrated polymer matrix allows selective permeability for small molecules while excluding larger fouling agents, thereby reducing clogging compared to porous fabrics.^15^ Additionally, hydrogels can be printed or moulded into complex geometries, enabling modular reactor designs and integration into advanced water treatment systems.^16^ PVA hydrogels are biocompatible, non-toxic, elastic/stretchable, and highly water-retentive, creating a stable microenvironment that sustains catalytic activity over extended periods.^17–19^ Furthermore, hydrogel encapsulation not only enhances stability but also maintains continuous water flow, making these nanocomposites highly suitable for advanced wastewater treatment.^20^

Conventional water treatment methods, such as chlorine-based disinfection, can generate harmful disinfection byproducts (DBPs).^21^ In contrast, Advanced Oxidation Processes (AOPs), which rely on hydroxyl radicals (˙OH), offer an efficient method for degrading organic pollutants and are effective in removing phenol from wastewater.^22,23^ Catalytic wet peroxide oxidation (CWPO), a type of AOP, uses catalysts to activate hydrogen peroxide and generate ˙OH under mild conditions, providing an environmentally friendly alternative.^24^ Iron-based catalysts, including those used in the Fenton reaction, are common but produce iron sludge and require acidic conditions and elevated temperatures.^25–28^ To overcome these limitations, alternative catalysts such as nickel and cobalt have been investigated; however, poor stability hinders their reuse. Gold nanoparticles (AuNPs), including supported variants, have shown high catalytic efficiency over a broad pH range and under mild conditions.^28,29^ For instance, AuNPs on hydroxyapatite achieved 80% phenol conversion at 343 K in 2 hours with a H_2_O_2_ : phenol ratio of 500 : 1.^27^ Supports such as TiO_2_, Fe_2_O_3_, and activated carbon further enhance the performance of AuNPs, offering high total organic carbon (TOC) conversion and minimal metal leaching.^27,30^ Catalyst morphology and surface area are key to activity, and recent studies highlight the potential of low-dimensional nanomaterials to improve catalytic performance further.^31–34^

In this study, we assess AuNTps for the degradation of phenolic compounds under ambient conditions. We begin by considering the reduction of 4-nitrophenol, a typical example used to evaluate new nanomaterials, before examining the degradation of phenol. After considering the materials free in suspension, we then assess micron-thick, freestanding, inkjet-printed AuNTp/PVA-hydrogel composites. Such printed composites would allow for easy application, recovery, and reuse of the catalyst, as well as offer potential for applications in bioelectronics and wound dressings.

## Experimental

### Materials

Gold (III) chloride trihydrate (520 918), 4-nitrophenol (>99%, 241 326), and poly (vinyl alcohol) (PVA; Mw 146–148 kDa; 99%+ hydrolysed), (PVA; 89 kDa – 98 kDa, 99+ hydrolysed), (PVA; 30–70 kDa, 87–90% Hydrolysed), PVA MOWIOL® 4–98 (27 kDa, 98.0–98.8% hydrolysed) and dimethyl sulfoxide (DMSO) (LC-MS grade, 85 190) were purchased from Sigma Aldrich. Phenol, hydrogen peroxide (30% w/v), hydroquinone (99.5%, 10 313 292), benzoquinone (99%, 10 638 463) and sodium borohydride (0 210 289 425) were purchased from Fisher Scientific. Catechol (F005090) was purchased from Fluorchem. Trisodium citrate (45 556) and methyl orange (17 874) were purchased from Alfa Aesar. Purified horseradish peroxidase (HRP) (Rz = 3.47) was purchased from Thermo Scientific. The HRP enzyme, as determined by the pyrogallol method performed by the supplier, had a specific activity of 254 U mg^−1^ enzyme (one unit catalyses the production of 1 mg of purpurogallin from pyrogallol in 20 seconds at 20 °C and pH 6.0). All chemicals were of analytical grade and used without further purification. Polyethylene terephthalate (PET) with a film thickness of 0.1 mm was purchased from Goodfellow. Ultrapure water (Millipore Milli-Q) with a resistivity of 18.2 MΩ cm at 25 °C was used in all experiments.

### Synthesis of AuNPs and AuNTps

AuNPs were synthesised using the Turkevich method.^35^ Briefly, 4 mL of 50 mM HAuCl_4_ was mixed with 196 mL of Milli-Q water in a round-bottomed flask, heated to boiling, and stirred. Subsequently, 10 mL of 77.6 mM sodium citrate was added. The solution turned ruby red, and after 20 minutes, the heating was stopped. The solution was rapidly cooled by placing the flask in an ice bath, allowing it to reach room temperature (23 °C) within 10 minutes, and then transferred to 50 mL Falcon tubes. The AuNPs were collected by centrifugation at 4600*g* for 90 minutes. Following the removal of the supernatant, the AuNP pellets were resuspended in 5 mL of Milli-Q water. This resuspension and centrifugation process was repeated twice. The final pellets were combined and stored for future use.

For the synthesis of AuNTps, 4 mL of 0.21 mM methyl orange, 1 mL of 5 mM gold chloride, and 500 µL of 100 mM sodium citrate were added sequentially to a glass vial, at room temperature (23 °C) and left overnight (17 hours). The product was collected and rinsed three times by centrifugation at 3000 g for 60 minutes, then resuspended in MQ water for further use.

### Characterisation of AuNPs and AuNTps

Absorption spectra were obtained using an Agilent Cary 5000 spectrophotometer with samples placed in Brand Micro UV cuvettes (10 mm path length). Transmission electron microscopy (TEM) images were obtained using a Tecnai G2 Spirit TEM (T12) operated at an acceleration voltage of 120 kV, equipped with a Gatan US4000 CCD camera for image capture. Scanning TEM (STEM) based electron energy loss spectroscopy (EELS) was acquired on an SU-9000EA (Hitachi HighTech) scanning electron microscope equipped with a cold field emission gun electron source (∼0.4 eV full width at half maximum in energy spread), a high angle annular dark field (HAADF) STEM detector, and an integrated energy loss spectrometer (Hitachi HighTech). The microscope was operated at 30 keV beam energy. EELS data were processed using HyperSpy (version 1.7.2).^36^ The spectra were aligned to the zero loss peak to sub-pixel precision, followed by removal of intensity spikes arising from X-rays or other high-energy photons arriving at the detector. EELS spectrum images were analysed by non-negative matrix factorisation (NMF) as implemented within HyperSpy. Atomic force microscopy (AFM) height measurements were conducted to determine the thickness of the AuNTps. A drop of AuNTps solution with a concentration (optical density at 400 nm) OD_400_ = 0.25 was placed on a freshly cleaved plasma-treated mica disc and dried overnight. The next day, samples were imaged in the air at room temperature in “Tapping mode” with FastScan-A probes (nominal resonant frequency of 1.4 MHz and spring constant of 18 N m^−1^) on a Bruker Dimension FastScan Bio AFM running NanoScope software version 9.4. Scanning parameters were a scan size of 1000 nm, with a resolution of 1024 samples/line.

The particle size distribution of AuNPs and the area analysis of AuNTps were analysed from TEM images using ImageJ software. Atomic absorption spectroscopy (AAS), employing an Agilent 240 fs atomic absorbance spectrophotometer, was used to determine the Au content of AuNPs and AuNTps dispersions with various optical densities in aqueous suspensions.

### Reduction of 4-nitrophenol with AuNPs and AuNTps

For the reduction of 4-nitrophenol (4-NP) with AuNPs and AuNTps, 10 µL of 15 mM 4-NP was combined with 980 µL of 20 mM sodium borohydride and then mixed with 10 µL of a 900 µg mL^−1^ AuNX (where X = P or T) dispersion in a cuvette with a path length of 1 cm. The temperature was maintained at 20 °C. Control measurements were taken in the absence of the Au nanomaterial. The reaction progress was monitored by collecting UV-Vis spectra at 1-minute intervals, and the reaction kinetics were evaluated by analysing the optical absorbance at 400 nm. Rate constants were determined using a pseudo-first-order kinetics model, as described by eqn (1):1
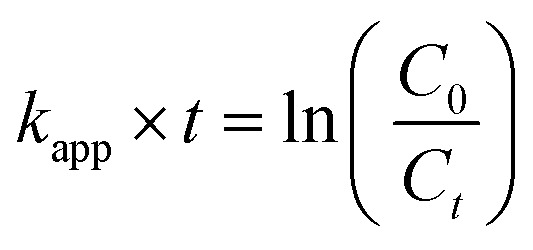
where *k*_app_ represents the apparent rate constant, *t* is the reaction time, and *C*_0_ and *C*_*t*_ denote the initial concentration (obtained from the absorbance) of 4-NT at *t* = 0 and its concentration at time *t*, respectively. The apparent rate constant, *k*_app_, was determined by applying a Box-Lucas fit to the plot of Ln (*C*_0_/*C*_*t*_) *versus* time, eqn (2).

2 *y* = *a*(1 − *e*^−*bx*^)

The mass normalised rate constant *k*_1_ (min^−1^ g^−1^) was calculated by dividing *k*_app_ by the mass of the catalyst.

### Oxidation of phenol by hydrogen peroxide in the presence of AuNX/HRP catalysts

For the oxidation of phenol with AuNTps or AuNPs, the reaction mix consisted of a total of 1 mL of aqueous suspension containing 500 µL of acetate buffer (pH = 3.5), 100 µL of 900 µg mL^−1^ AuNX, 50 µL of H_2_O_2_ (9.8 M), and 25 µL of 10 mM phenol. For the oxidation of phenol with HRP, a total of 1 mL of reaction mix consisting of 500 µL of phosphate-buffered saline, PBS (pH = 5.6), 10 µL of 1 µM HRP, 50 µL of hydrogen peroxide, H_2_O_2_ (9.8 M), and 25 µL of 10 mM phenol was used. The reactions correspond to a phenol to H_2_O_2_ ratio of ∼1 : 2000. For a phenol to H_2_O_2_ ratio of 1 : 500, 50 µL of 2.5 M H_2_O_2_ was used.

### HPLC for the detection of phenol

High-performance liquid chromatography (HPLC) analysis was performed using an Agilent 1290 Infinity II HPLC system (Agilent, Santa Clara, CA, United States) with a diode array detector. Chromatographic separations were performed using an Agilent InfinityLab Poroshell 120 EC-C18 (2.1 × 50 mm, 1.9 µm) at a column temperature of 40 °C. The mobile phase used was 0.1% TFA in water (95%) and 0.1% TFA in acetonitrile (5%), and the gradient ran to 5% water over 5 minutes at a flow rate of 0.5 mL min^−1^. The Diode Array Detector (DAD) recorded the chromatogram at a wavelength of 280 nm.

### Preparation of AuNX-PVA hydrogel cubes

AuNX-PVA hydrogel cubes were synthesised by dissolving 5 wt% PVA (Mw 146–148 kDa; 99%+ hydrolysed) in MQ water, heating the mixture at 100 °C with constant stirring until complete dissolution. The resulting transparent solution was transferred to a 10 mm × 10 mm × 35 mm cuvette, filling approximately one-quarter of its volume, and subjected to three freeze–thaw cycles (at −20 °C for 1 hour, followed by thawing at room temperature). The gels were extracted from the cuvette and stored in water.

### Preparation of AuNTp-PVA hydrogel tiles

AuNTp-PVA hydrogel tiles were prepared by dissolving 10 wt% PVA (27 kDa) in an AuNTps suspension, heating at 90 °C with continuous stirring until fully dissolved. The cooled suspension was mixed with DMSO to constitute one-third of the total volume, resulting in a final optical density (OD_400_) of 10. It was then poured into a polytetrafluoroethylene mould (10 mm × 10 mm × 2 mm). The total AuNTps concentration in the gel was estimated to be 9 µg. Subsequently, the mould/gel was frozen at −20 °C for 1 hour and thawed at room temperature three times. Finally, the gels were removed from the mould and stored in water.

### Inkjet printing of AuNX-PVA hydrogel meshes

To prepare a 30 mL ink solution containing 4 wt% AuNX-PVA with a water/DMSO ratio of (2/1), 4 wt% PVA (27 kDa, 98.0–98.8% hydrolysed) was dissolved in a 20 mL aqueous suspension of AuNX. The concentration of AuNX stock was adjusted to achieve an optical density of 2 at 400 nm. This mixture was heated to ∼100 °C on a hot plate with continuous stirring until the PVA granules were completely dissolved. After cooling, 10 mL of DMSO was added to the AuNX-PVA suspension and stirred for 30 minutes at room temperature. The resulting AuNX-PVA ink was filtered through a 0.2 µm cellulose filter and stored for later use. The inks were used within three days of formulation; if kept longer, they tended to form a gel, resulting in nozzle blocking.

AuNX-PVA hydrogels were printed using a Dimatix materials printer 2850, operating in drop-on-demand mode with a piezoelectric printhead containing 16 nozzles (21 µL diameter, 10 pL drop volume). PET film with a thickness of 0.1 mm was purchased from Goodfellow and used as the printing substrate. It was pre-cleaned with isopropyl alcohol and MQ water and dried with N_2_. Due to the polymeric nature of the ink, effective droplet formation occurred solely at a cartridge temperature of 60 °C. The printer plate temperature was maintained at 60 °C to ensure optimal droplet spreading on the PET substrate and to aid the drying of the printed PVA layers. Printing parameters were also optimised for droplet ejection and pattern formation. The Dimatix model waveform used the following settings: meniscus level of 5, nozzle voltage of 27 V, and maximum jetting frequency of 5 kHz. Fabrication of mesh structures involved printing a designated number of layers, each consisting of four parallel lines, each measuring 10 mm × 1 mm with a 1 mm gap, followed by a 30-minutes drying period. An additional set of the same layered lines was then printed in an orthogonal direction. Pattern resolution was 1693 dpi, corresponding to 15 µm drop spacing. Print head angle adjusted to 3.4° based on drop spacing. After printing, the pattern was dried for 30 minutes on the printer platen heated at 60 °C, and the mesh was subsequently peeled from the PET support and stored in a Petri dish under moist conditions for further use.

Before peeling off from the PET support, the thickness of the printed lines was measured using the Dektak XT 2D contact profilometer, which features a 2 µm tip and a force of 3 mg.

### Viscosity and surface tension measurements

Rheological measurements of different concentrations of PVA (Mw 146–148 kDa; 99%+ hydrolysed and PVA (27 kDa, 98.0–98.8% hydrolysed) were performed using an Anton Parr MCR 302 rheometer with a parallel plate setup. Comprising two parallel plates (the top plate with a diameter of 25 mm), with a rotating top plate where torque was applied. The fluid was confined between these plates due to capillary forces, with a 0.5 mm gap between the parallel plates. The viscosities were measured at shear rates up to 1000 s^−1^, *i.e.* towards the lower range of those experienced during inkjet printing (10^3^ to 10^6^ s^−1^).

The surface tension of various PVA formulations was determined using the pendant drop technique using the OCA 15 EC from Data Physics Instruments. The PVA suspensions were dispensed using a syringe DS-D 1000 SF fitted with a disposable dosing needle with controlled liquid dosing (∼5 µL). Measurement and analysis were performed using the dpiMAX software.

### Catalytic assessment of printed AuNTp-PVA hydrogel mesh for the reduction of 4-NP

The catalytic efficiency of AuNTps integrated inkjet-printed PVA meshes and drop-cast gels was evaluated by reducing 4-nitrophenol using NaBH_4_, maintaining the same reactant concentrations as in the suspension-based reaction. The meshes were placed in cuvettes containing the reaction mixture. Due to their lightweight and thin design, the gels floated, positioning the catalytic surface above the UV-spectrometer beam path. For reusability tests, the meshes were retrieved using tweezers, rinsed in Milli-Q water, and transferred to fresh reaction mixtures.

### Oxidation of phenol using printed AuNTp-PVA meshes

The reaction was carried out according to the procedure employed for solution-based phenol oxidation, maintaining the same reagent concentrations, with AuNTps incorporated into printed meshes. A total reaction volume of 2 mL was divided into 500 µL aliquots. Two AuNTp-PVA meshes (printed with AuNTps at OD_400_ = 10) were placed in 500 µL of the phenol reaction mixture. After the specified reaction time, the meshes were removed using tweezers, and the solution was analysed by HPLC to determine the residual phenol content. For reusability tests, the meshes were rinsed with acetate buffer before being transferred to fresh reaction mixtures.

## Results and discussion

### Synthesis and physical characterisation of 1D gold nano tapes (AuNTps) and spherical gold nanoparticles (AuNPs)

**Fig. 1:**
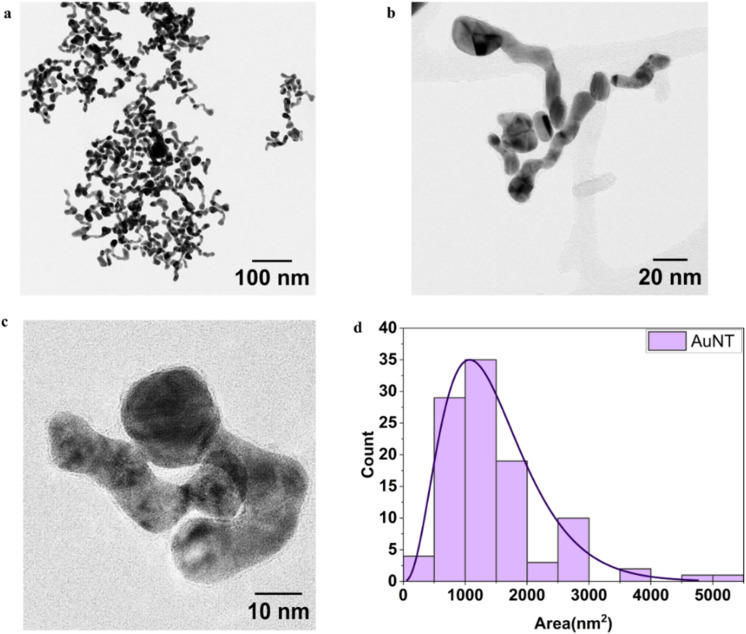
(a) TEM image of AuNTps at a scale of 100 nm; (b) higher-magnification TEM image at 20 nm scale; (c) high-resolution TEM image at 10 nm scale, highlighting individual AuNTps with characteristic head–tail morphology; and (d) histogram illustrating the area distribution of AuNTps, obtained from TEM images and analysed using ImageJ. The solid line overlaid on the histogram represents a Gamma distribution fit applied to model the asymmetric size distribution of the AuNTps.

To evaluate the catalytic properties of AuNTps relative to AuNPs, we first characterised their structural and morphological features. The quasi-1D gold nanotapes (AuNTps) are of particular interest as nanozymes or catalysts due to their high surface-to-volume ratio and abundance of exposed edge sites. AuNTps were synthesised in a one-step aqueous approach at 20 °C, using methyl orange as a confining agent. More conventional spherical AuNPs were used as a comparison and were synthesised using the Turkevich method.^35^ The morphology of the synthesised AuNTps (Fig. 1) and AuNPs were assessed using transmission electron microscopy (TEM). AuNTps displayed a “tadpole” structure, characterised by a thicker spherical or hexagonal “head” and a quasi-1D ''tail”. Analysis using ImageJ estimated the average surface area per AuNTp to be 1470 ± 860 nm^2^ (*N* = 103). Additional TEM images of AuNTps, highlighting the head and tail structures, are presented in Fig. 1(b) and (c). Detailed area calculations of these structures are provided in the SI. The formation of AuNTps is proposed to follow a multi-step pathway similar to that previously reported for larger 2D gold sheets.^5^ First, Au(iii) ions in HAuCl_4_ associate with aromatic rings of the methyl orange (MO), reduction by SC generates primary Au nuclei. These nuclei subsequently grow into small nanoflakes, with their upper/lower dimensions confined by interactions with MO molecules. To minimise high surface energy, these nanoflakes undergo oriented attachment, selectively joining along side facets. Larger nanoflakes then act as secondary nucleation sites, enabling further attachment of freshly formed nanoflakes. This hierarchical assembly ultimately yields extended tape-like structures with distinct head and tail regions, as observed in TEM. We note in the absence of the MO quasi-spherical gold nanoparticles are formed wih a UV peak circa 50 nm and absorbance near 530 nm.

The TEM image of the AuNPs, shown in Fig. S1, verifies the uniform distribution of the AuNPs, revealing an average diameter of 11.5 ± 0.7 nm (*N* = 200). Assuming a spherical shape, this corresponded to a surface area per particle of ∼415 ± 52 nm^2^. For an equivalent mass of gold, AuNTp possess a surface area four times larger than that of AuNPs.

**Fig. 2:**
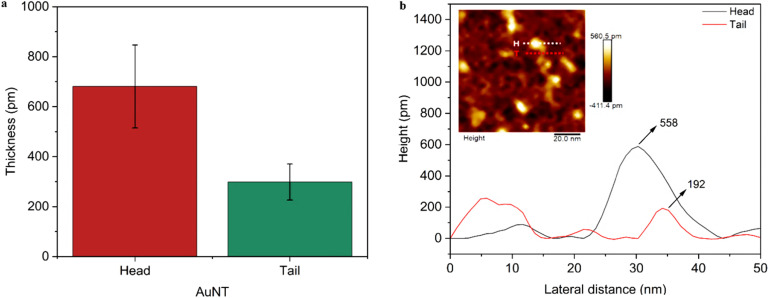
AFM analysis of AuNTps on a mica substrate. (a) A quantitative comparison of head and tail thickness (mean ± SD, *N* = 25). (b) Representative height profiles illustrate the head–tail morphology, accompanied by AFM images (insets) and line profile across a sample region indicating multiple features of varying heights that indicate the regions of higher head thickness (white) and thinner tails (red). Corresponding peak heights are labelled for clarity.

Atomic force microscopy (AFM) analysis revealed a distinct head–tail morphology in the AuNTps. The head region exhibited an average thickness of 681 ± 165 pm (*N* = 25), corresponding to approximately three atomic layers of gold. In contrast, the thinner, two-dimensional tail measured 298 ± 72 pm (*N* = 25), which—considering the uncertainty—falls within the range of a single atomic layer (Fig. 2(a)). These measurements support the structural heterogeneity observed in the nanostructure as observed in the TEM characterisation. A representative image and corresponding height profiles are shown in Fig. 2(b). Further sample images and height analysis are provided in Fig. S2. AFM analysis further emphasised the significant structural asymmetry of the Au nanotapes, showing that ‘he’ ‘ead’ region is consistently thicker and more elevated than ‘he’ ‘ail’. This morphological heterogeneity was considered when estimating the molar concentration of the AuNTps and comparing them to standard spherical AuNPs.

For the AuNP suspension at OD_400_ = 1 with a 90 µg mL^−1^ concentration, the volume of a single AuNP is approximately 7.9 × 10^−19^ cm^3^, giving a mass of 1.54 × 10^−17^ g and about 4.7 × 10^4^ atoms per particle. Hence, the number of AuNPs in 90 µg is calculated as 5.8 × 10^12^, which corresponds to a molarity of ∼10 nM. For AuNTps, using an average thickness (average of head and tails) of 0.49 nm and an area of 1470 nm^2^, the volume of a single AuNTp is 7.19 × 10^−19^ cm^3^, with a mass of 1.39 × 10^−17^ g, and each containing ∼4.2 × 10^4^ atoms. At the same concentration, this yields 6.5 × 10^12^ AuNTp with a molarity of ∼11 nM. Depending on whether only the head (681 pm) or tail (298 pm) thickness is used, the molarity ranges from 7.74 nM to 17.68 nM.

**Fig. 3:**
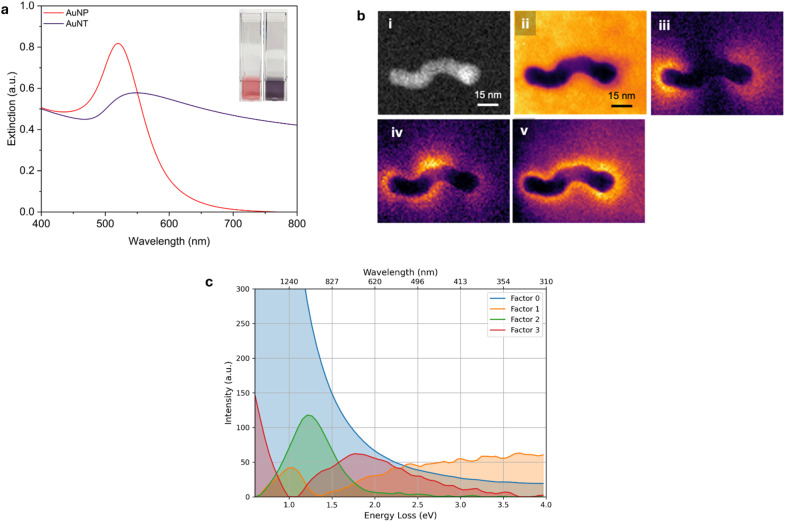
(a) UV-Vis extinction spectra of AuNPs (red) and AuNTps (purple), normalised at 400 nm; insets show respective dispersions in Milli-Q water. (b) STEM EELS spectral image dataset of AuNTps with (i) HAADF image and surface plasmon modes, (ii) zero loss peak (factor 0), (iii) dipole (factor 2), (iv) quadrupole (factor 3), (v) transverse and intraband (factor 1), and (c) spectral maps corresponding to spectral mode.

The UV-Vis absorption bands of AuNTps and AuNPs are shown in Fig. 3(a). AuNPs solutions synthesised, *via* the Turkevitch method, displayed a characteristic bright red colour with a sharp plasmon band centred at 520 nm, consistent with their uniform size and shape. This is a characteristic of localised surface plasmon resonance in isotropic particles. In contrast, the AuNTps dispersion had a deep purple colour associated with a broad peak centred on 534 nm but which extends into the near-IR, reflecting (i) a heterogeneity in the dimensions of the individual tapes and (ii) multiple plasmonic resonances associated with the length, width, and height of the tapes (tail and head regions). This geometry affects the plasmonic behaviour; the thicker head region accommodates lower-energy, delocalised plasmon modes, while the thinner tail promotes higher-energy, more confined modes.^37^ The presence of thicker ‘head’ regions (∼0.7 nm) and thinner ‘tails’, corresponding to only 2–5 atomic layers, confirms the atomically thin nature of AuNTps.

The plasmonic modes of the AuNTps were investigated using EELS in the STEM. AuNTps were prepared on graphene support films in this case, and so the dielectric medium contributions to the resonance energies are expected to be slightly shifted to those for dispersions in water. The full EELS spectrum image dataset (a spectrum at every position in a 2D scan) was simplified into a set of maps and corresponding spectral factors using Non-negative Matrix Factorisation (NMF). NMF has been used widely to automatically extract mode distributions and spectral components from plasmonic metal nanoparticles probed by EELS.^38–41^Fig. 3(b(i)) shows the HAADF-STEM image of an isolated AuNTp of length 52 nm. Electrons passing through the sample with no or negligible energy losses contribute to what is termed the zero-loss peak. The tail of the zero-loss peak was separated from the sample-specific energy losses (Fig. 3(b(ii))), factor 0 by NMF. Fig. 3(b(iii) and (iv)) represents surface plasmon resonance modes along the length of the AuNTp, assigned as the dipolar (*m* = 1) and quadrupolar (*m* = 2) longitudinal modes typical of both straight^42,43^ and bent^44^ plasmonic nanorods as well as similar asymmetrical “nanocarrot” geometries.^45^ These modes are characterised by standing wave patterns in the surface charge distribution with m nodes. In turn, they correspond to distinct resonance mode energies (Fig. 3(c)). The dipolar plasmon resonance occurs at ∼1.2 eV, and the quadrupolar mode occurs at ∼1.8 eV. The NMF spectral factor for the quadrupolar mode is incompletely separated from the zero-loss peak tail, giving it a residual rise at low energies.^31,32^Fig. 3(b(v)) corresponds to factor 1 in the energy spectra and corresponds to transverse modes (dipoles perpendicular to the long axis of the AuNTp) and interband transitions in Au.^38,46–48^ There is some asymmetry in the EELS maps of these features with the long-axis modes showing greater intensity at the ‘tail’ and the transverse modes showing greater EELS intensity at the ‘head’, possibly due to difference in the thickness and asymmetry of the structure though contributions from differences in the local dielectric environment arising from the support film or from varying thickness of surrounding amorphous carbon (ligand shell) cannot be ruled out. Table S1 summarises the EELS spectra of AuNTps with the main plasmon modes identified, compared with the corresponding broadened features in the UV-Vis spectrum. These results suggest that the dipolar modes of AuNTps of different lengths likely dominate the broadened UV-Vis spectra, with some contribution also from the transverse modes and quadrupolar modes excited by plane wave light for oblique orientations of the long axis. The UV-Vis and EELS results demonstrate that the observed plasmonic features are sensitive to changes in shape, providing clear evidence of morphological variation.

### Catalytic and enzymatic properties of AuNTps

**Fig. 4:**
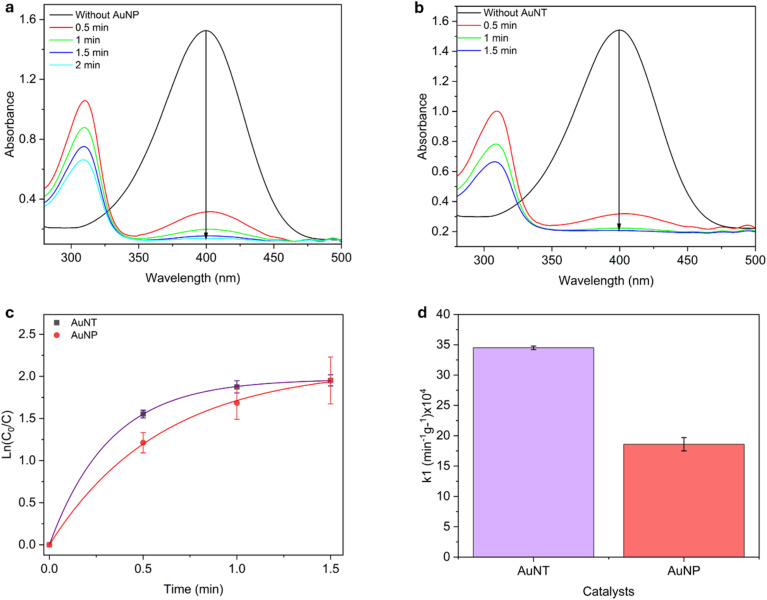
UV-Vis absorption spectra showing the catalytic reduction of 4-nitrophenol to 4-aminophenol over 2 minutes. (a) and (b) show the reaction of nitrophenol with NaBH4in the presence of 9 µg of AuNTps and AuNPs, respectively. (c) Shows the plot of ln(*C*_0_/*C*_*t*_) *versus* time used to calculate the apparent rate constant (*k*_app_), and (d) presents the comparison of mass-normalised rate constants (*k*_1_) for the AuNTp and AuNP catalyst. Error bars represent the standard deviation from three independent measurements performed on a total sample volume of 1 mL for each condition.

The reduction of 4-NP by NaBH_4_ has become a well-established reaction to assess the catalytic activity of noble metal nanocatalysts.^49–52^ Before evaluating the catalytic activity of AuNTps within printed hydrogels, their activity was first assessed in a free suspension form. 4-NP solution initially exhibits a light-yellow colour, but upon adding NaBH_4_, it transitions to a bright yellow, indicating the formation of 4-nitrophenolate (*l*_max_ = 400 nm) (Fig. S3(a)). This compound was subsequently reduced to 4-aminophenol (4-AP) (*l*_max_ = 300 nm) *via* the addition of the catalyst (AuNTps or AuNPs) (Fig. S3). Without a catalyst, the peak at 400 nm remained unchanged throughout the 10-minutes monitored period (Fig. S3(b)). When 9 µg AuNTps were added, the peak at 400 nm disappeared within a minute, whereas with AuNPs, the reaction took nearly 2 minutes (Fig. 4(a) and (b)) for an equivalent mass of gold. The apparent rate constant, *k*_app_, was determined by fitting the plot of ln (*C*_0_/*C*_*t*_) *versus* time (Fig. 4(c)), where *C*_0_ and *C*_*t*_ denote the initial concentration (obtained from the absorbance) of 4-nitrophenol at time *t* = 0 and at time *t*, respectively. The values obtained were 3.10 ± 0.03 min^−1^ for AuNTps and 1.7 ± 0.1 min^−1^ for AuNPs. The mass normalised rate constant *k*_1_ calculated for AuNTps was 34.5 × 10^4^ min^−1^ g^−1^, compared to AuNPs (18.6 × 10^4^ min^−1^ g^−1^) (Fig. 4(d)). This demonstrates that AuNTps are nearly twice as catalytically efficient as AuNPs for 4-NP reduction on a per-mass basis. The estimated *k*_1_ value for the AuNPs was consistent with the previously reported values of 22 × 10^4^ min^−1^ g^−1^ for 4 nm sized AuNPs and 6 × 10^4^ min^−1^ g^−1^ for 16 nm sized AuNPs.^53^ A rough estimate of the area for the AuNTps is nearly 3.5 times that of AuNPs for the same amount of gold by weight. Thus, the enhanced activity in the AuNTps is most likely attributed to the increased surface area, which provides abundant surface and edge atoms.

### Phenol oxidation with AuNTps/AuNPs/HRP

**Fig. 5:**
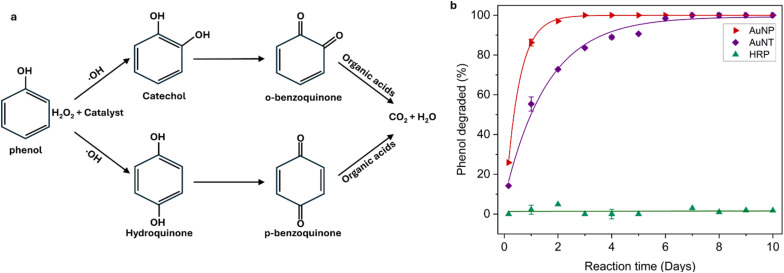
Oxidation of phenol with hydrogen peroxide (H_2_O_2_) at room temperature at a phenol: H_2_O_2_ ratio of 1 : 2000. (a) The proposed reaction pathway of phenol oxidation shows intermediates and final products. (b) HPLC analysis of phenol degradation using AuNTps, AuNPs, and HRP as catalysts in suspension. Error bars represent the standard deviation from three independent measurements performed on a total sample volume of 1 mL for each condition.

The oxidation of phenol by hydrogen peroxide in the presence of a catalyst primarily involves two pathways, as shown in Fig. 5(a), involving intermediates such as catechol, hydroquinone, and benzoquinone. Subsequent attack by ˙OH radicals leads to ring opening, the formation of organic acids and the production of CO_2_ and H_2_O as end products.^25,27,54^ UV-Vis monitoring was complicated by H_2_O_2_ interference (Fig. S4 and S5), so HPLC was used to quantify phenol degradation.

Reactions were run with 90 µg mL^−1^ AuNTps/AuNPs or 10 nM HRP under acidic conditions (pH 3.5 for Au; pH 5.6 for HRP). Reactions were conducted for each catalyst using phenol: H_2_O_2_ ratios of 1 : 2000 and 1 : 500. Since most industrial wastewater is acidic, the pH of the reaction mixture was adjusted to 3.5 with acetate buffer. A highly acidic or alkaline pH is unfavourable to HRP, so the pH of the phenol solution with HRP was adjusted to 5.6 using PBS buffer, which falls within the optimal activity range of HRP.^18^ Controls showed negligible phenol degradation at room temperature without a catalyst. Literature comparisons typically involve higher catalyst loadings (up to 7 g L^−1^), elevated temperatures (80 °C), or harsher conditions.^24,26,27,29^

The oxidation of phenol was investigated using H_2_O_2_ in the presence of AuNTps, AuNPs and HRP as catalysts (Fig. 5(b)). At a phenol: H_2_O_2_ ratio of 1 : 2000, AuNPs achieved rapid degradation, with nearly 26% phenol removal within the first 4 hours (day 0) and complete degradation in 6 days. Under the same conditions, AuNTps exhibited slower kinetics, degrading about 14% within 4 hours and reaching ∼90% degradation within 7 days. Control experiments carried out without catalysts showed negligible phenol removal (Fig. S6(b)), confirming that H_2_O_2_ alone is insufficient to drive the reaction effectively. At an equivalent molar concentration (10 nM), horseradish peroxidase (HRP) displayed minimal activity, with less than 10% degradation over 10 days, in sharp contrast to the gold-based catalysts. For comparison, experiments performed at a lower H_2_O_2_ ratio (1 : 500) are presented in the SI (Fig. S6(c)). Under these conditions, phenol oxidation proceeded more slowly, confirming that the availability of H_2_O_2_ is a limiting factor in the catalytic cycle, with higher concentrations enabling more efficient degradation. To assess whether HRP activity could be improved at higher concentrations, its loading was increased to 2 µM. Under these conditions, HRP completely degraded phenol within 4 hours (Fig. S6(d)). While this shows that HRP can be highly effective, it highlights the need for enzyme concentrations several orders of magnitude higher than those of Au catalysts to achieve comparable performance.

The formation of phenol oxidation intermediates (catechol, hydroquinone, and benzoquinone) was quantified by HPLC (Fig. S7). At a phenol: H_2_O_2_ ratio of 1 : 2000, both AuNPs and AuNTps showed transient benzoquinone formation (∼25–33% at day 0), which decreased to negligible levels by day 10, while catechol and hydroquinone remained very low. At the lower ratio of 1 : 500, side-product formation was minimal, consistent with slower overall degradation. These results confirm that efficient oxidation at 1 : 2000 proceeds with rapid conversion of intermediates to final products (CO_2_ and H_2_O).

To date, only one reported study has demonstrated phenol degradation at room temperature using gold-based catalysts—specifically, a sunlight-assisted Fenton system employing gold nanoparticles supported on diamond. While effective, that approach requires both external energy input and costly support materials, and does not confirm complete mineralisation to CO_2_ and H_2_O, instead focusing on the formation of biodegradable intermediates.^31^ In contrast, the present system achieves phenol oxidation under ambient conditions without light activation, relying solely on the intrinsic peroxidase-like activity of the nano catalysts to activate hydrogen peroxide. These findings highlight the superior efficiency of nanomaterial-based catalysts compared with natural enzymes, supporting their continued development as potent alternatives for oxidative degradation processes. Interestingly, AuNTps and AuNPs exhibited similar performance in phenol oxidation, despite differing in their ability to reduce 4-NP. This suggests that the catalytic oxidation of phenol may involve a different rate-limiting step or mechanistic pathway that is less dependent on the surface geometry or facet structure of the catalyst. Unlike the surface-sensitive electron transfer in 4-NP reduction, phenol oxidation likely proceeds through bulk-generated hydroxyl radicals from H_2_O_2_ activation, which may interact similarly with both spherical and anisotropic particles.

### AuNX-PVA hydrogels

**Fig. 6:**
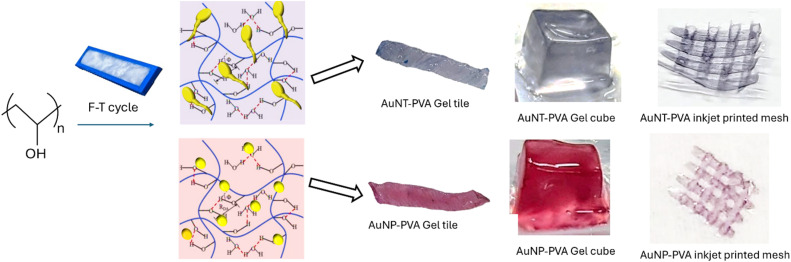
Digital image of AuNTp- and AuNP-PVA hydrogel tiles and cubes prepared using the repeated freeze–thaw method with 140 kDa PVA. The tiles and cubes were formed using moulds, while micro-thin mesh structures were created *via* inkjet printing using an ink formulated with 27 kDa PVA and an AuNX concentration corresponding to OD_400_ = 2.

Following the successful room temperature degradation of phenol using AuNTps and AuNPs, the AuNTps were incorporated into PVA hydrogels, creating a biocompatible 3D matrix for catalyst attachment to enable reusability and minimise nanoparticle loss. Fig. 6 illustrates the process of loading AuNTps (dark purple) and AuNPs (red) into PVA (140 kDa) hydrogels using the freeze–thaw method, utilising different moulds. The hydrophilic nature of the gel should allow the ready diffusion of water-soluble molecules into the matrix and reaction at the catalytic sites. Our goal in developing inkjet-printable PVA-AuNTp hydrogels was to reduce the gel from the millimetre to the micron scale while retaining the gold loading to improve diffusion times for analytes to reach catalytic sites. However, high-molecular-weight PVA has proven unsuitable for inkjet printing due to its high viscosity.

Changing the solvent from pure water to a mixture of water and DMSO, using a lower-molecular-weight PVA (30–70 kDa), and a DOH content of 87–90% showed good printability at up to 10 wt%. Unfortunately, such polymers could not be gelled post-printing (even by increasing the PVA content to 20 wt%).^55^ Detailed comparisons of PVA types and gelation outcomes are provided in Tables S2 and S3. Ultimately, PVA (27 kDa) at 4 wt% in water/DMSO produced printable and gelable inks. The uniform colour distribution observed in the printed PVA hydrogel structures indicates that the AuNTps were evenly dispersed in the precursor inks, with little nanoparticle clumping prior to printing. Image analysis of grayscale intensity across the printed strands (Fig. S18) further confirms macroscopic uniformity. Small differences in grayscale levels are caused by optical effects, such as local variations in reflectivity or differences in lighting, rather than by variations in nanoparticle concentration. Although the AuNTp distribution within the ink itself is very uniform, differences in the final printed features are likely to occur due to wetting/drying behaviour inherent to inkjet printing, including local thickness changes or uneven solvent evaporation. Whilst outside the scope of this work, understanding the distribution of the particles within such printed gels *via* FIB/TEM merits further study.

Viscosity and interfacial property measurements identified 4 wt% PVA (Mw 27 kDa) in a 2 : 1 water/DMSO mixture as the optimal ink formulation. This concentration maintained a viscosity below 10 mPa s across 20–60 °C, exhibited a surface tension of 57.0 mN m^−1^, and yielded a *Z* number of 6.9, all within the operational window for inkjet printing. Higher PVA loadings (>8 wt%) exceeded the viscosity threshold and compromised jetting, while the solvent composition rather than the polymer concentration dominated the surface tension behaviour. A 4 wt% PVA ink was successfully printed, exhibiting stable jetting without nozzle clogging (Fig. S8–S11). The deposited structures gelled effectively post-printing to form uniform hydrogel patterns with adequate mechanical integrity.

The aqueous stability and leaching behaviour of AuNTp-PVA hydrogels were evaluated using UV-Vis spectroscopy over 7 days. Spectra collected directly through the gel at early time points (1 min, 1 h, 2 h) and again on day 7 showed minimal variation, indicating strong retention of AuNTps within the PVA matrix (Fig. S15(a) and (b)). To further assess leaching, control experiments were conducted by suspending gel tiles in Milli-Q water without obstructing the beam path. No characteristic AuNTp absorbance (500–700 nm) was detected in the surrounding water over 2 hours or after 7 days, even after gel swelling and settling (Fig. S15(c) and (d)). These results confirm negligible leaching of AuNTps into the aqueous phase, demonstrating that the nanoparticles remain stably entrapped within the hydrogel network.

### Inkjet printing of PVA-AuNX hydrogel meshes

**Fig. 7:**
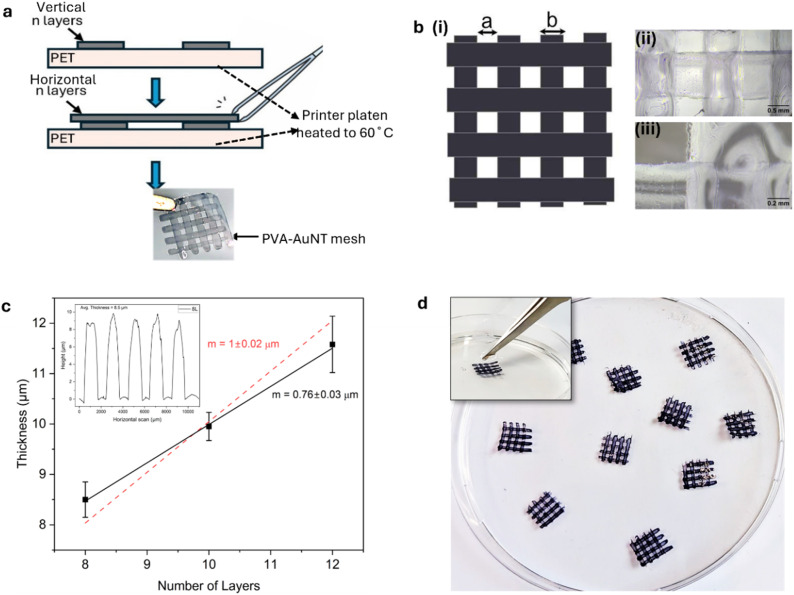
(a) Inkjet printing process for mesh structures with PVA hydrogel, showing freestanding hydrogels. (b) (i) Final mesh pattern alongside optical microscopy images of the printed PVA hydrogel mesh, captured using (i) 2× and (ii) 10× objective lenses. (c) A graph showing the variation in horizontal printed lines with thickness measured across the lines with increasing layers (n) was measured using a Dektak with an inset showing a sample measurement of 8 layers (top left). Error bars represent the standard deviation from three independent measurements for each data point. (d) Image of inkjet-printed AuNTp-PVA hydrogel meshes fabricated using an ink with AuNTp concentration at OD_400_ = 10. The top-left inset shows the mesh retaining its shape upon immersion in water, demonstrating structural stability.

Ink containing AuNTps at an optical density of 2 (measured at 400 nm) was used to print mesh structures with 1 mm spacing onto PET films and then dried (Fig. 7). Optical microscopy confirmed consistent, well-formed lines and junctions. The linear fit of thickness *versus* number of layers yielded a slope of 0.76 ± 0.03 µm per layer with a *y*-intercept of 2.4 µm (*R*^2^ = 0.997), indicating an anomalously thick first layer. If the fit is instead forced through the origin, the slope increases to ∼1.0 µm per layer (*R*^2^ = 0.91), but with reduced accuracy. This behaviour is consistent with the “first-drop effect” in inkjet printing, where the initial deposited layer spreads more extensively, resulting in a greater thickness than subsequent layers. A similar trend can be inferred from the data of Monne *et al.*, who reported a thickness of 5.68 µm for four layers.^16^ If their first layer is assumed to be anomalously thick (*e.g.*, ∼3 µm, comparable to our 2.4 µm), then the remaining three layers correspond to ∼0.89 µm per layer, which is in close agreement with our measured slope. This suggests that despite differences in polymer type and printing conditions, both studies reveal comparable per-layer growth once the effect of the first layer is accounted for. High OD inks (OD_400_ = 5 and 10) were also successfully printed, enabling tunable gold loading. Fig. 7(d) shows stable mesh structures retaining shape in water.

### Reduction of 4-NP to 4-AP in AuNX-PVA catalysts

**Fig. 8:**
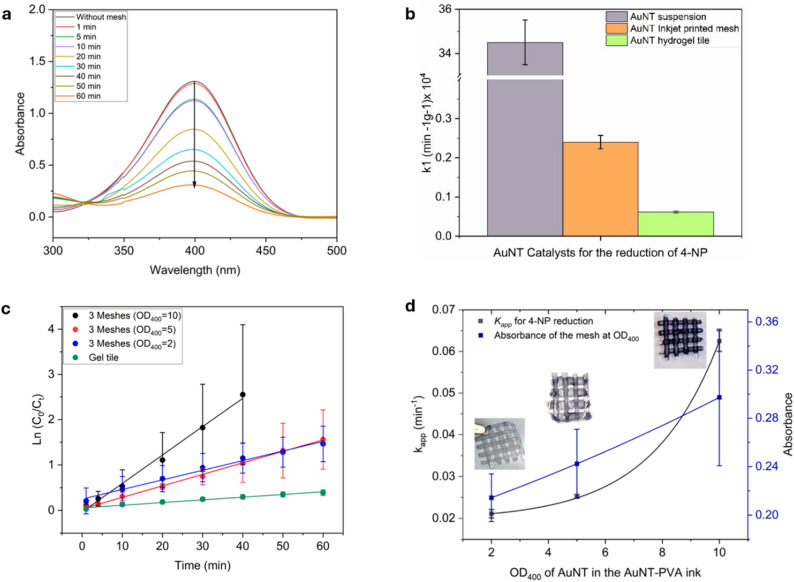
Evaluation of the catalytic performance of AuNTp-PVA hydrogel meshes and AuNTp-PVA hydrogel tiles, each containing 9 µg of AuNTps, in the reduction of 4-Nitrophenol (4-NP) to 4-Aminophenol (4-AP). (a) UV-Vis absorption spectra recorded over 60 minutes with AuNTp-PVA mesh (OD_400_ = 2). (b) Mass-normalised rate constants *k*_1_ for AuNTps in suspension, inkjet-printed meshes and gel tiles. (c) Plot of Ln (*C*_0_/*C*_*t*_) *versus* time for *k*_app_ calculation for meshes with AuNTps concentrations at OD_400_ = 10, 5, and 2, and gel tiles with 9 µg AuNTps (OD_400_ = 2). (d) The apparent rate constant, *k*_app_, is plotted as a function of gold loading. Error bars indicate the standard deviation calculated from three independent measurements for each data point.

To enable catalyst reuse and improve handling, AuNTps were embedded in PVA hydrogels as both drop-cast tiles and inkjet-printed meshes. The mesh design, owing to its reduced thickness and open architecture, was expected to enhance diffusion and reagent access to the catalytic sites. To highlight the advantages of inkjet-printed mesh structures over thicker PVA hydrogel tiles, we compared both formats using the same gold content. Fig. 8(a) shows that the meshes achieved ∼75% 4-NP reduction in 60 minutes, compared to just 30% for the gel tiles (Fig. S22(a)). Unsurprisingly, both formats showed slower reaction rates than freely suspended AuNTps (Fig. 8(b)). The mass-normalised rate constant dropped from 34.5 × 10^4^ min^−1^ g^−1^ for free AuNTps to 0.24 × 10^4^ min^−1^ g^−1^ for meshes and 0.066 × 10^4^ min^−1^ g^−1^ for tiles. Rate constants were calculated from Ln (*C*_0_/*C*) plots using equal amounts of AuNTps (9 µg), shown as blue (meshes) and green (tiles) circles in Fig. 8(c).

The reduced activity of gel-based AuNTps compared to free AuNTps is mainly due to diffusion-limited kinetics, as they are immobilised rather than freely dispersed. This is supported by the ∼10-fold higher activity of meshes over tiles, a limitation that may be less relevant in flow-through applications. Despite slower kinetics, both mesh and tile hydrogels offer easy catalyst recovery and consistent reuse performance (Fig. S22(b)). To further optimise performance, mesh catalysts with higher AuNTps loadings (OD_400_ = 5 and 10) were tested. As shown in Fig. 8(c and d). OD_400_ = 5 meshes showed little improvement over OD_400_ = 2, suggesting that diffusion rather than gold content was limiting. However, OD_400_ = 10 achieved complete reduction within ∼30 minutes, with *k*_app_ ∼0.06 min^−1^, three times faster than OD_400_ = 2 or 5 (Fig. S23(a) and (b)).

### Phenol oxidation with AuNTp-PVA hydrogels

Inkjet-printed AuNTp-PVA hydrogels (OD_400_ = 10) were tested for catalytic phenol oxidation using H_2_O_2_. Four mesh structures were used per 1 mL reaction and compared against free AuNTps under identical conditions. HPLC results (Fig. S24(a)) showed that only 26% of phenol was degraded within 4 hours using the mesh, with no significant improvement over 2 or 4 days, *versus* 100% degradation over 7 days in suspension. However, the mesh was reusable, achieving consistent performance across three separate reactions (Fig. S24(b)). The reduced activity of immobilised AuNTps likely arises matrix-induced suppression of catalytic sites or restricted H_2_O_2_ access. Matrix-induced effects refer to structural and chemical constraints imposed by the hydrogel network, including steric hindrance that partially blocks active sites, altered microenvironment (pH and polarity), and reduced reagent accessibility within dense regions of the gel. To mitigate diffusion constraints, inkjet printing was implemented to fabricate thin, freestanding mesh structures with micron-scale thickness, enhancing fluid access to the catalyst while enabling macroscopic handling and reusability. To assess whether diffusion remains a significant barrier, the phenol diffusion times within the PVA hydrogel were estimated. Reported diffusion coefficients for small aromatic molecules in PVA range from 10^−10^ m^2^ s^−1^ for loose/swollen gels to 10^−12^ m^2^ s^−1^ for dense, highly cross-linked gels. For a 10 µm-thick printed mesh of moderate density (*D* ≈ 10^−11^ m^2^ s^−1^), phenol would need to diffuse approximately 5 µm to reach the innermost AuNTps. Using *t* ≈ *L*^2^/*D*, the diffusion time is ∼2.5 s, increasing to ∼25 s for the gel tiles. These timescales are negligible compared to the overall reaction duration (hours to days), indicating that while diffusion may contribute to reduced activity, it is unlikely to be the primary limiting factor compared to matrix-induced effects. Despite the lower efficiency compared to catalysts in suspension, the printed hydrogel meshes offer significant advantages in catalyst handling, recovery, and potential device integration. We observed that printed PVA meshes maintained structural integrity for approximately 4 days, whereas bulk PVA gels remained intact for over a week, suggesting that mechanical stability should be considered in future optimisation.

## Conclusion

This study presents a robust and scalable strategy for integrating high-activity gold nanozymes into reusable catalyst formats suitable for environmental remediation under ambient conditions. AuNTps, owing to their anisotropic morphology and high surface area, showed superior catalytic efficiency in 4-NP reduction and effective phenol oxidation. Notably, AuNTps were nearly twice as catalytically efficient as AuNPs for 4-NP reduction on a per-mass basis, emphasising the impact of morphology on surface-sensitive electron–transfer reactions. In contrast, morphology-driven effects were less pronounced in phenol oxidation, which likely proceeds *via* bulk-generated hydroxyl radicals and is less dependent on facet structure. This suggests that the rate-limiting step in phenol oxidation may be governed more by radical generation and diffusion rather than direct electron transfer at specific surface sites. However, both the AuNPs and the AuNTps proved effective for the room temperature oxidation of phenol to CO_2_ and H_2_O.

To enable easy recovery and reuse, AuNTps were incorporated into PVA hydrogels, forming composite materials that retained catalytic function while improving handling. These hydrogels were further enhanced by inkjet printing into thin, micrometre-scale mesh structures, thereby improving reagent diffusion and increasing access to catalytic sites. Inkjet-printed AuNTp-PVA meshes achieved a nearly fourfold increase in catalytic rate over drop-cast AuNTp gel tiles for 4-NP reduction (0.24 × 10^4^*vs.* 0.07 × 10^4^ min^−1^ g^−1^), while maintaining structural integrity and reusability. For phenol oxidation, printed meshes achieved 26% degradation in 4 hours at room temperature and retained activity across multiple reuse cycles, compared to complete degradation in 7 days for suspended AuNTps. The reduced activity is attributed to diffusion limitations and matrix-induced suppression of catalytic sites, highlighting the need to optimise hydrogel–nanoparticle interactions. Overall, this work establishes a versatile route for fabricating printable nanozyme composites with potential applications in portable water purification systems and real-time pollutant sensing platforms.

## Author contributions

The manuscript was written through the contributions of all authors. All authors have approved the final version of the manuscript. N. J. carried out the methodology, investigation, data curation, formal analysis, visualisation, and writing – original draft. S. D. E. contributed to conceptualisation, validation, interpretation of results, supervision, writing and review of the manuscript. K. C. assisted with supervising and reviewing the manuscript. S. C. and Q. R. performed the EELS experiments and contributed to the analysis and interpretation of the EELS data.

## Conflicts of interest

There are no conflicts to declare.

## Abbreviations

AuNTps: gold nanotapes
PVA: polyvinyl alcohol
4-NP: 4-nitrophenol
DBPs: disinfection byproducts
AOP: advanced oxidation process
OH˙: hydroxyl radicals
CWPO: catalytic wet peroxidation
AuNPs: gold nanoparticles
AC: activated carbon
AuNS: gold nanosheets
TMB: 3,3′,5,5′-tetramethylbenzidine
DAB: 3,3′-diaminobenzidine
DMSO: dimethyl sulfoxide
HRP: horseradish peroxidase
PET: polyethylene terephthalate
TEM: transmission electron microscopy
AFM: atomic force microscopy
OD_400_: optical density at 400 nm
UV-vis: ultraviolet-visible spectroscopy
AAS: atomic absorption spectroscopy
PBS: phosphate-buffered saline
H_2_O_2_: hydrogen peroxide
HPLC: high-performance liquid chromatography
DOH: degree of hydrolysis
4-Aminophenol: 4-AP

## Supplementary Material

NA-008-D5NA00968E-s001

## Data Availability

The data associated with this paper are available from the University of Leeds at https://doi.org/10.5518/1728. Supplementary information (SI) is available. See DOI: https://doi.org/10.1039/d5na00968e.
