# Supplementary material for: Atomically thin gold embedded in inkjet-printed PVA hydrogels: flexible catalysts for ambient phenol degradation
Source: Nanoscale Adv. 2026 Jan 12;8(5):1626–40. doi: 10.1039/d5na00968e (PMC12869187; doi:10.1039/d5na00968e)
Supplement: NA-008-D5NA00968E-s001 [file NA-008-D5NA00968E-s001.pdf]

Supplementary Information (SI) for Nanoscale Advances.
This journal is © The Royal Society of Chemistry 2026

Atomically Thin Gold Embedded in Inkjet-Printed PVA Hydrogels: Flexible Catalysts for Ambient Phenol Degradation

Nizzy James,[a] Sean Collins,[c,d], Quentin Ramasse,[b,c] Kevin Critchley[a] and Stephen D. Evans[a*]

*Corresponding author,E-mail: s.d.evans@leeds.ac.uk

[a.] *School of Physics and Astronomy, Bragg Centre for Materials Research,, University of Leeds, Woodhouse Lane, LS2 9JT Leeds, United Kingdom.*
[b.] *School of Physics and Astronomy and School of Chemical and Process Engineering, University of Leeds, Woodhouse Lane, LS2 9JT Leeds, United Kingdom*
[c.] *SuperSTEM Laboratory, SciTech Daresbury Campus, Keckwick Lane, WA4 4AD Daresbury, United Kingdom*
[d.] *Bragg Centre for Materials Research, School of Chemical and Process Engineering and School of Chemistry, University of Leeds, Woodhouse Lane, LS2 9JT Leeds, U.K.*

Area calculation OF AuNTps from TEM images

To analyse the area of gold nanoparticles (AuNTps), the image was opened in the ImageJ application. A scale bar was added to the image before the area calculations were performed. To enhance contrast, the image was converted to grayscale by selecting *Image > Type > 8-bit*. Next, the threshold was adjusted to distinguish the AuNTps from the background by choosing Image > Adjust > Threshold and adjusting the sliders until the nanoparticles were clearly highlighted. To measure the area, the nanoparticles were selected, and then *Analyse> Tools > ROI Manager* was opened. A dialogue box appeared; *Show All* was selected, and the *Labels* boxes were enabled. Using the *Wand Tool*, each nanoparticle area in the image was selected by clicking on it. The areas were displayed in the ROI Manager window, and the results were copied to an Excel sheet. Since this method only calculated the area of one side of the nanoparticles, the results were multiplied by 2 to determine the final total area.

Characterisation of AuNPs with TEM

Figure S1 shows the morphological analysis of gold nanoparticles (AuNPs) obtained from Transmission Electron Microscopy (TEM), including size distribution and high-resolution structural details.

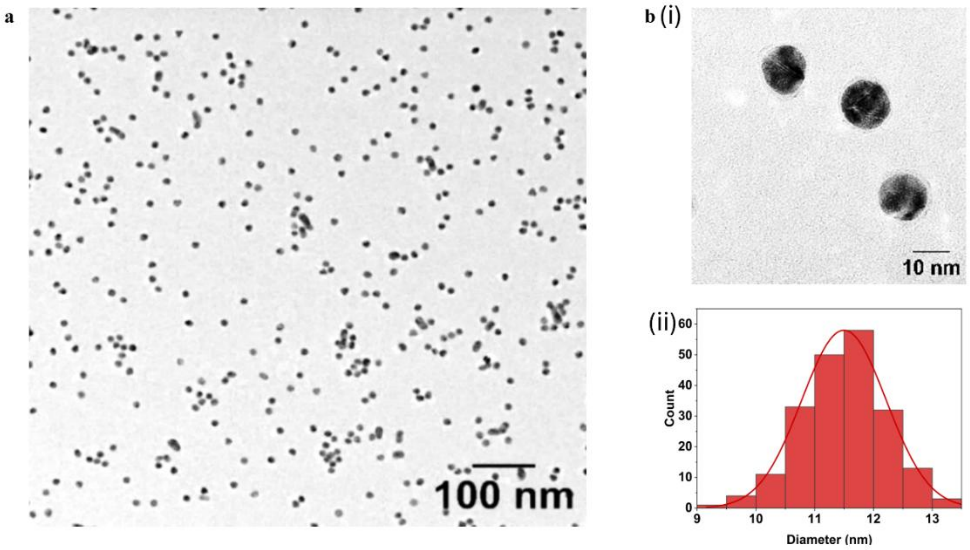

Figure S1 TEM image of AuNPs: (a) at a scale of 100 nm; (b-i) high-resolution image at 10 nm scale; (b-ii) particle diameter histogram.

AFM of AuNTps

Figure S1 presents the atomic force microscopy (AFM) analysis of gold nanotapes (AuNTps), providing topographical insights into their morphology, including surface features and height distribution.

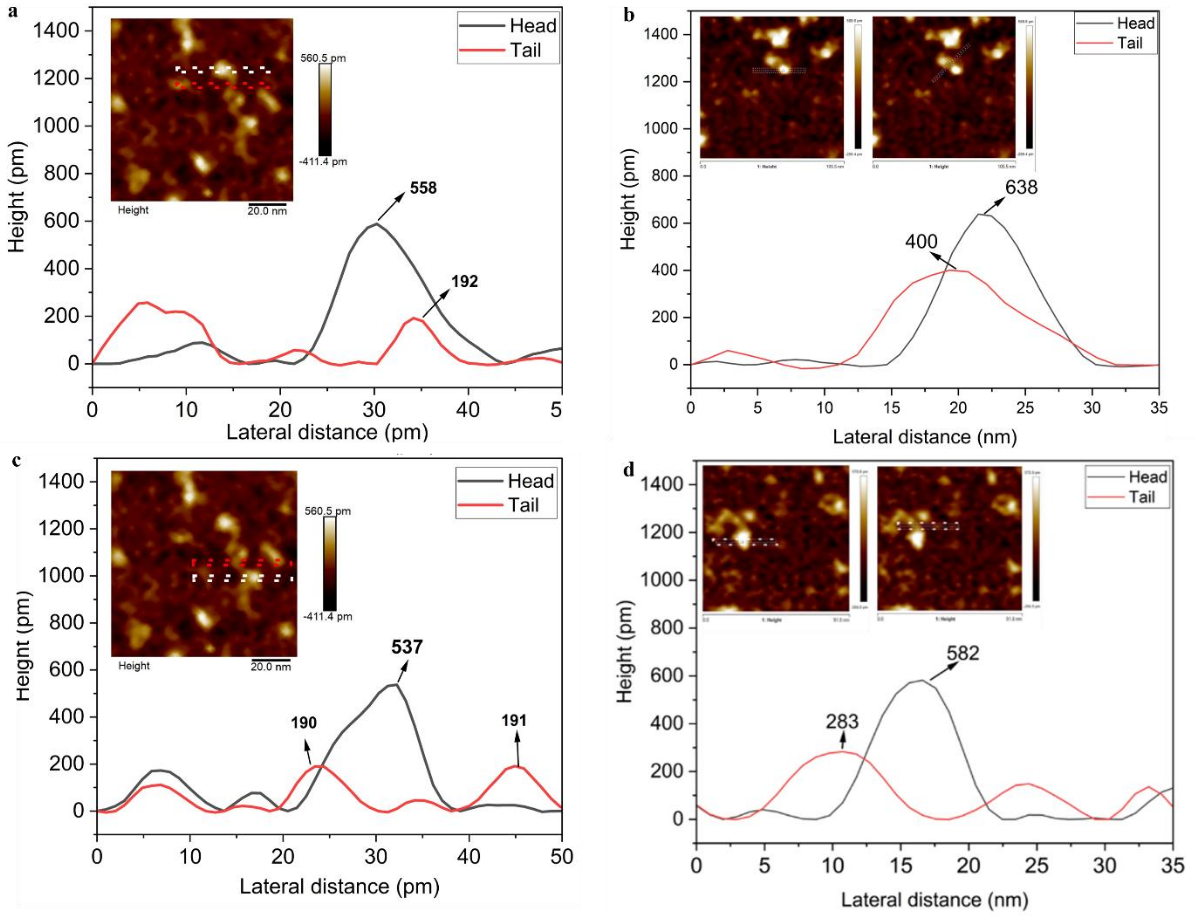

Figure S2 Section analysis of AFM images of AuNTp: (a–d) Line profiles taken across selected regions, showing variations in height across different features. Insets display the AFM topography images with the corresponding section lines marked.

EELS of AuNTp

Table S1 Summary of EELS factors of AuNTp showing main plasmon modes. Wavelengths correspond to the top axis (λ≈1240/E).

**Factor**	**Peak (eV)**	**Peak wavelength (nm)**	**Peak max Intensity (a.u.)**	**Notes**
**Factor 0 (ZLP tail)**	0 (ZLP)	—	—	Zero-loss peak tail; not a plasmon resonance; decays with energy. Residual contributions may overlap with low-energy plasmon signals.

**Factor 1 + (Transverse interband)**	~1.10 (sharp); Broad band ~1.5–3.8	~1127 (sharp); Broad band starting at 827	~35 (sharp); ~60 (broad)	A small, sharper peak is observed at ~1.1 eV; a broad transverse/interband band spans 1.5–3.8 eV, resulting from transverse modes and Au interband transitions. In UV-Vis, this contributes to the broad shoulder near 500–600 nm.
**Factor 2 (Longitudinal dipole)**	~1.35	~919	~120	Longitudinal dipole mode (m=1). Sharp and strong in the EELS of single AuNTp. In ensemble UV-Vis, this mode primarily contributes to the broad near-IR tail and does not appear as a sharp peak due to morphological heterogeneity and interparticle coupling.
**Factor 3 (Longitudinal quadrupole)**	~1.75	~709	~65	Longitudinal quadrupole mode (m=2). Well-resolved in EELS but overlaps partly with the ZLP tail. In UV-Vis, this feature merges into the broad visible extinction and cannot be isolated clearly.

Nitrophenol reduction using colloidal solutions

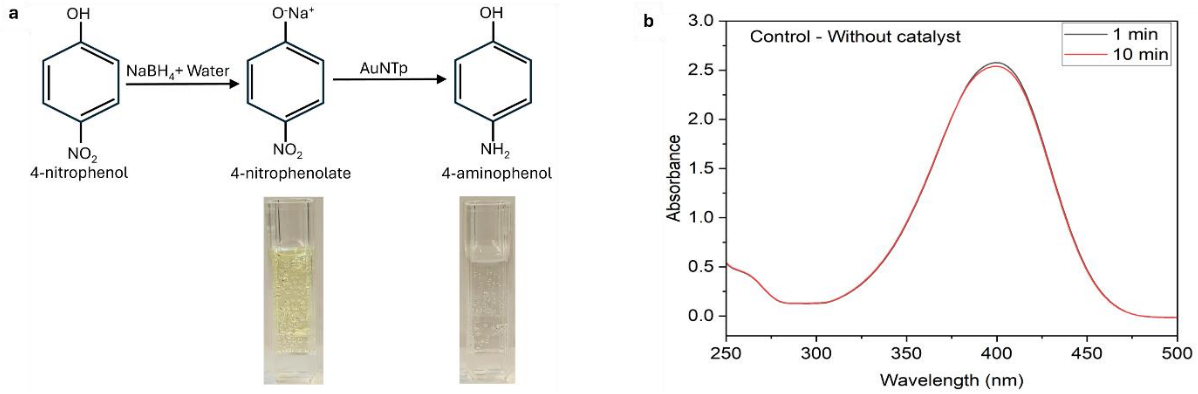

Figure S3 (a) Schematics showing the catalytic reduction process of 4-Nitrophenol (4-NP) to 4-Aminophenol (4-AP) with sodium borohydride ($NaBH_4$) in the presence of AuNTp (b) control without any catlaysts.

Phenol oxidation with AuNTp/AuNP/HRP

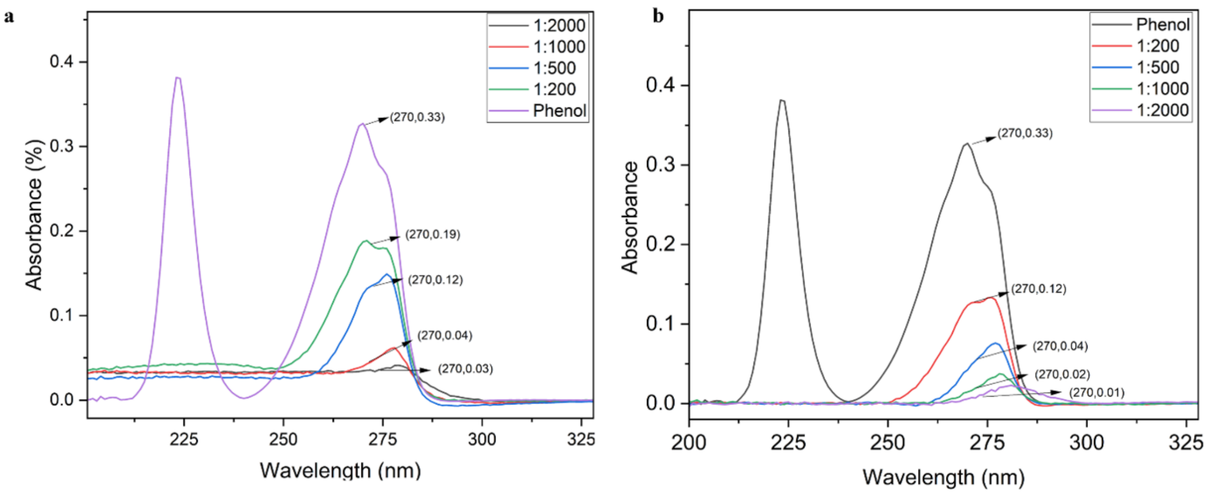

Figure S4 UV-Vis spectra illustrating the degradation/oxidation of phenol, monitored by the decrease in absorbance at ~270 nm, in the presence of varying H_2O_2:phenol ratios (1:2000, 1:1000, 1:500, and 1:20), catalysed by (a) AuNTp and (b) AuNP. Plateau or broadening around 270 nm—particularly at higher phenol: H_2O_2 ratios such as 1:2000 to 1:500—suggests possible interference or overlapping signals. Hydrogen peroxide shows strong absorbance in the 200–260 nm region, and at high concentrations, it can obscure or distort the phenol peak, making it appear less distinct or slightly shifted. This overlap can complicate the accurate interpretation of phenol degradation based solely on the 270 nm peak, especially during reactions involving excess H_2O_2.

HPLC analysis was performed for phenol: H_2O_2 molar ratios of 1:500 and 1:2000, monitoring phenol concentration over 10 days at room temperature.

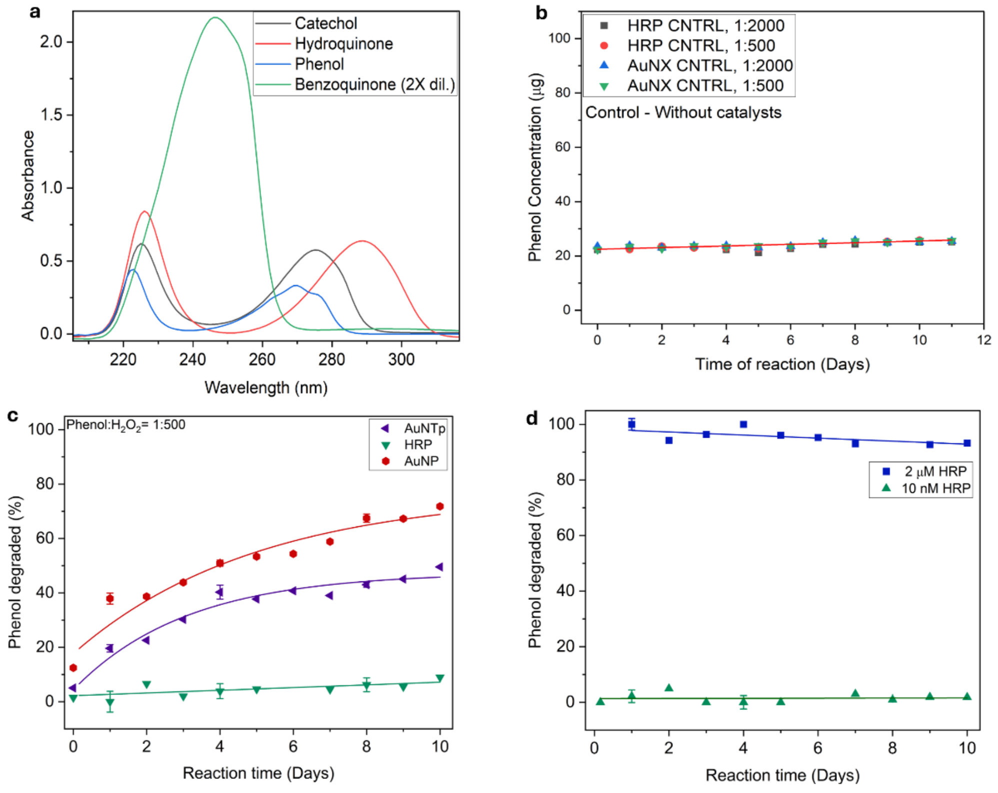

Figure S5 The oxidation of phenol with hydrogen peroxide (H_2O_2) using AuNTp, AuNP, and HRP as catalysts in suspension. (a) UV-Vis spectra of phenol and its reaction intermediates. (b) Control experiment without catalysts for different phenol: H_2O_2

ratios (1:2000 and 1:500) with HRP (in PBS buffer) and AuNP/AuNTp (in acetate buffer). (c) HPLC measurement of phenol degradation over 10 days with a Phenol: H_2O_2 ratio of 1:500 for AuNX (0.5 nM) and HRP (10 Nm). (d) Comparison of the activity of a higher concentration of HRP (2µM) for the oxidation of phenol with a phenol: H_2O_2 ratio (1:2000) over the activity of 10 nM HRP.

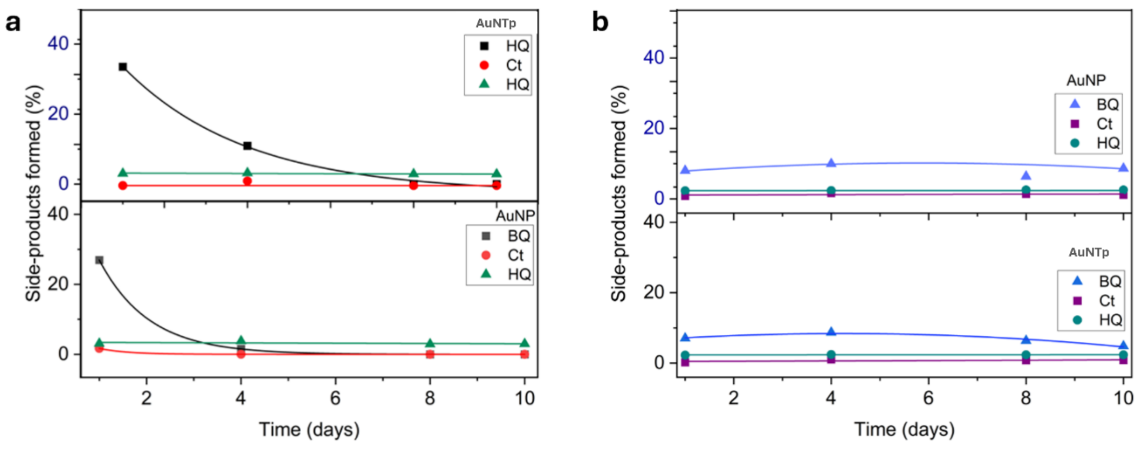

Figure S6 Oxidation of phenol with hydrogen peroxide: Quantification of side products such as benzoquinone (BQ), catechol (Ct), and hydroquinone (HQ) using HPLC for a (a) phenol: H_2O_2 = 1:2000 and (b) for a phenol: H_2O_2 = 1:500 with 90 µg of (a) AuNP (10 nM) and (b) AuNTp as catalysts at room temperature.

PVA-AuNX Ink formulation

Initially, various PVA formulations were evaluated to determine the most suitable options for inkjet printing. High-molecular-weight PVA grades consistently resulted in severe nozzle clogging, despite modifications to the formulation and adjustments to print parameters. This prompted a systematic investigation into the influence of the PVA's Molecular weight and DOH on printability and post-deposition gel formation. The assessments of gel formation for the various types of PVA tested are summarised in ***Tables S2 and S3.***

Table S2: *Checking gel formation of different concentrations of PVA of different types.*

PVA (kDa)	Degree of hydrolysis (%)	PVA Wt%	Solvents	Gel formation
140 -180	99	2	water	✔
			Water/DMSO: (4/1)	✔
			Water/DMSO: (2/1)	✔
89 – 98	99	2	water	✔
			Water/DMSO: (4/1)	✔
			Water/DMSO: (2/1)	✔

30 -70	87-90	2	water	✕
			Water/DMSO:(4/1)	✕
			Water/DMSO:(2/1)	✕
		4	Water	✕
		8	Water	✕
		10	Water	✕
		15	Water	✕
		20	Water	✕
MOWIOL® 4-98<br>27	98.0-98.8	2	Water	✕
		4	Water	✕
		8	Water	✕
		10	Water	✕
		15	Water	✔

A formulation using MOWIOL® 4-98, a low molecular weight PVA (27 kDa) with 98% hydrolysis at 15 wt%, facilitated gel formation after three freeze–thaw cycles. Table 2 compares PVA and MOWIOL with water-to-DMSO ratios of 4/1 and 2/1 for gel formation. A PVA (27 kDa) gel was observed after three freeze-thaw cycles with DMSO as a cosolvent at a 2:1 water-to-DMSO ratio and 8 wt% PVA. This was beneficial as it aimed to establish a gel with minimal PVA for effective jetting.

Table S3: *PVA- MOWIOL gel formation with Water/DMSO as solvent.*

PVA	Wt%	Solvent	Gel formation
MOWIOL® 4-98<br>27 kDa<br>98.0-98.8 mol%<br>hydrolysis	2	Water	✕
		Water/DMSO (4/1)	✕
		Water/DMSO (2/1)	✕
	4	Water	✕
		Water/DMSO (4/1)	✕
		Water/DMSO (2/1)	✕
	8	Water	✕
		Water/DMSO (4/1)	✕

		Water/DMSO (2/1)	Gel
	10	Water	✕
		Water/DMSO (4/1)	Gel
		Water/DMSO (2/1)	Gel
	15	water	✕
		Water/DMSO (4/1)	Gel
		Water/DMSO (2/1)	Gel

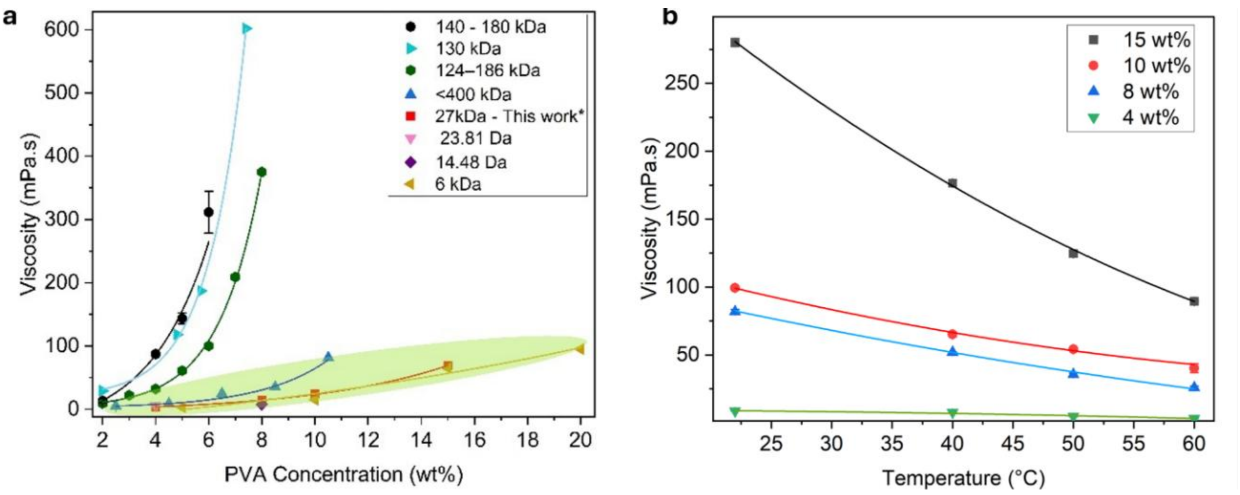

Figure S7 Comparison of the viscosity of PVA solutions with different molecular weights (140-180 kDa (checked),130 kDa,[1] 124-186 kDa,[2] <400 kDa,[3] 27 kDa (this work), 23.8 Da,[4] 14.48 Da,[4] 6 kDa[5]), highlighting previously reported inkjet-printable PVA types (in green shade), including the PVA (27 kDa) used in this study. (c) Variation in viscosity of PVA (27 kDa) suspensions in MQ water with temperature for different concentrations (4 wt%, 8 wt%, 10 wt%, and 15 wt%).

Sample viscosity measurements of polymer solutions in water/DMSO mixtures: effect of solvent ratio, temperature, and concentration.

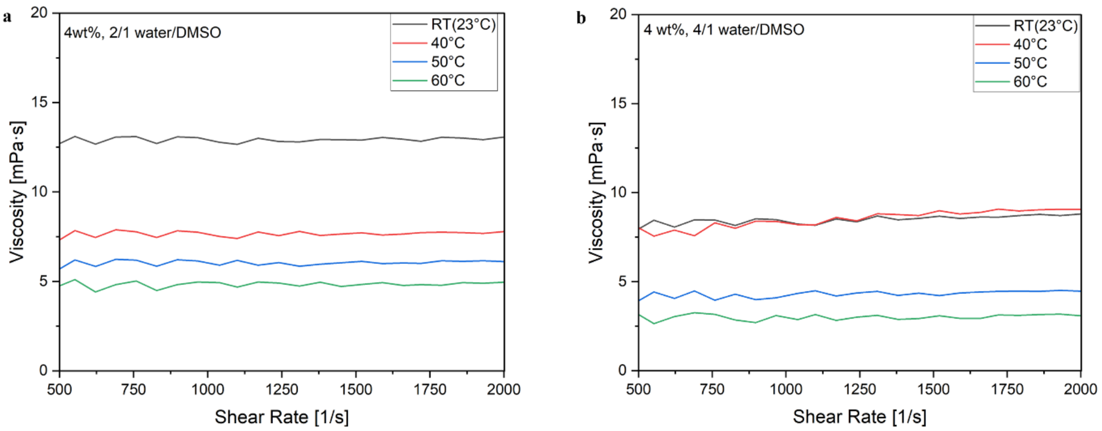

Figure S8 Viscosity as a function of shear rate for 4 wt% samples prepared in (a) 2/1 water/DMSO and (b) 4/1 water/DMSO solvent mixtures, measured at different temperatures (RT = 23 °C, 40 °C, 50 °C, and 60 °C). Both systems exhibit nearly Newtonian behaviour across the tested shear rate range, with viscosity decreasing as temperature increases.

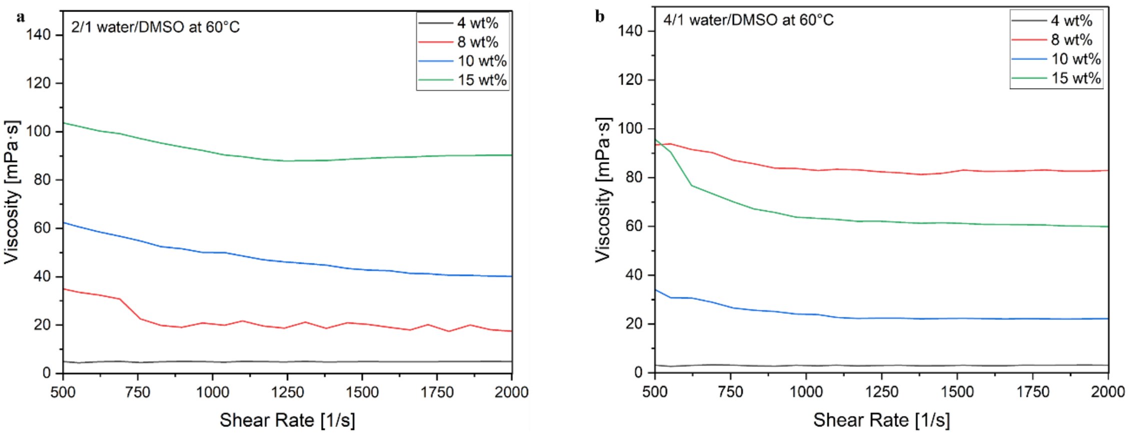

Figure S9 Viscosity as a function of shear rate for polymer solutions at different concentrations (4–15 wt%) in (a) 2/1 water/DMSO and (b) 4/1 water/DMSO solvent mixtures, measured at 60 °C. In both systems, viscosity increases with polymer concentration.

Interfacial tension measurements of polymer solutions in water/DMSO (4/1 and 2/1) mixtures at varying concentrations (4wt%, 8wt%, 10 wt% and 15 wt%).

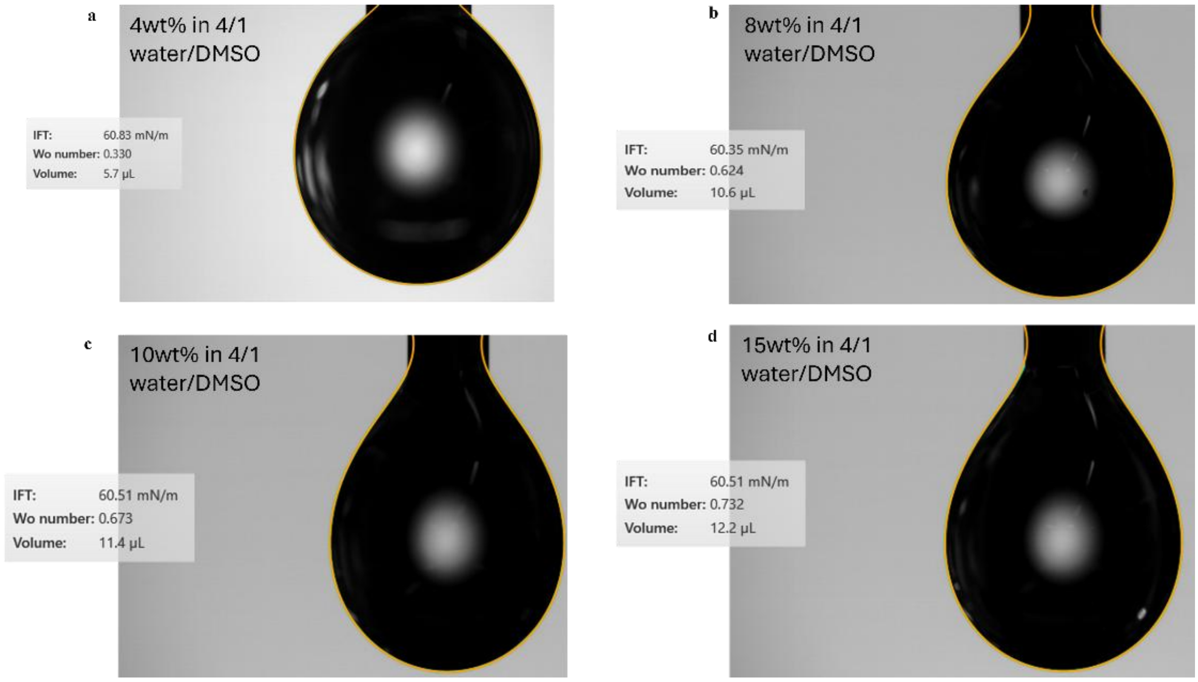

Figure S10 Pendant drop images of polymer solutions in a 4/1 water/DMSO solvent mixture at different concentrations (4–15 wt%). Interfacial tension (IFT), drop volume, and Wo numbers are indicated for each solution. The measured IFT values remain nearly constant (~60 mN/m) across the entire concentration range.

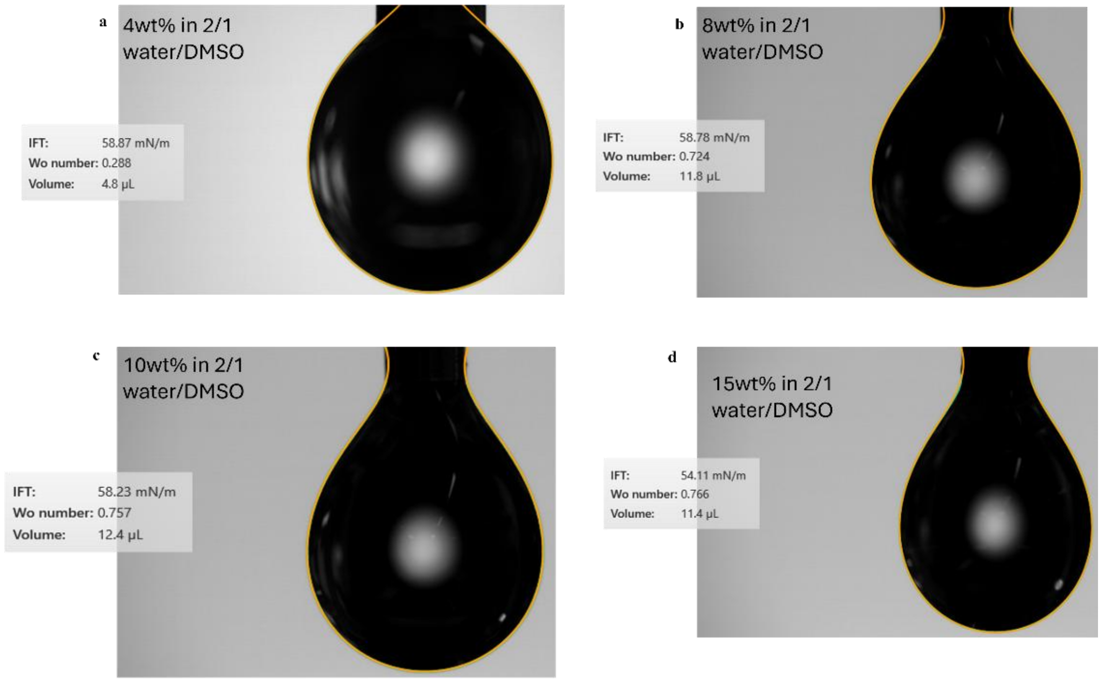

Figure S11 Pendant drop images of polymer solutions in a 2/1 water/DMSO solvent mixture at different concentrations (4–15 wt%). Interfacial tension (IFT), drop volume, and Wo numbers are indicated for each solution. The IFT values remain close to ~58 mN/m across most concentrations.

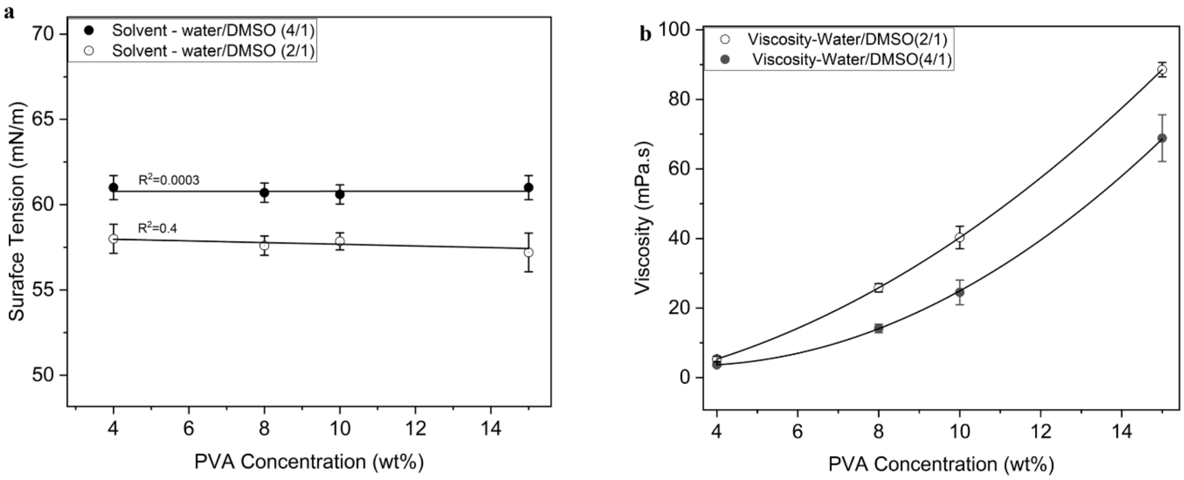

Figure S12 (a) Viscosity of PVA (27 kDa) solutions at 60 °C as a function of concentration for water/DMSO ratios of 4:1 and 2:1. (b) Surface tension of PVA (27 kDa) solutions with varying concentrations for water/DMSO ratios of 4:1 and 2:1.

Inkjet printing of PVA-AuNX meshes

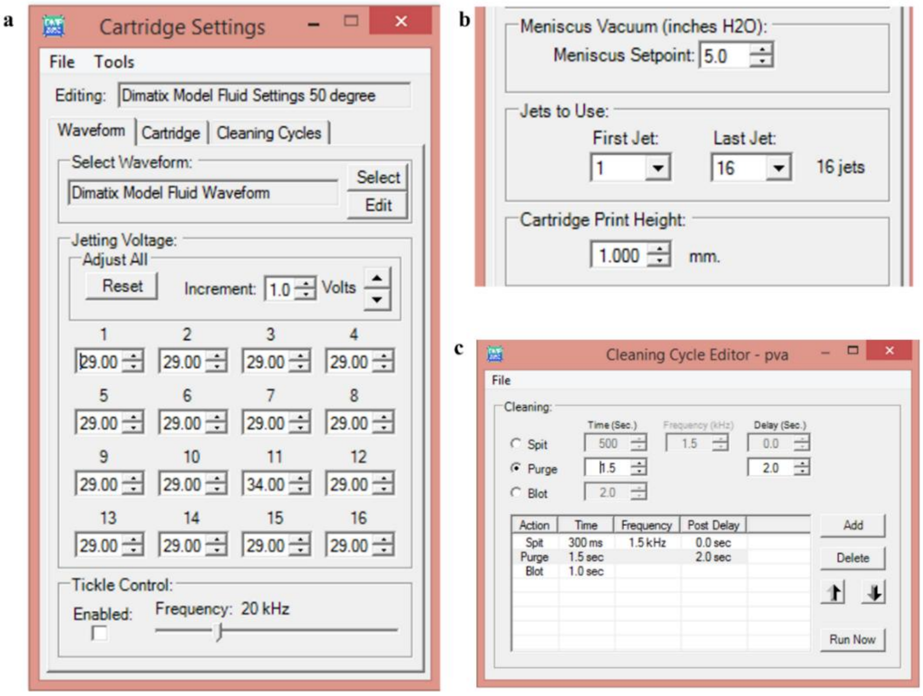

Figure S13 Cartridge settings for inkjet printing of a 4 wt% PVA solution in a 2/1 water/DMSO mixture include (a) voltage adjustments, (b) cartridge nozzle configurations for ink jetting, and (c) cartridge cleaning cycles.

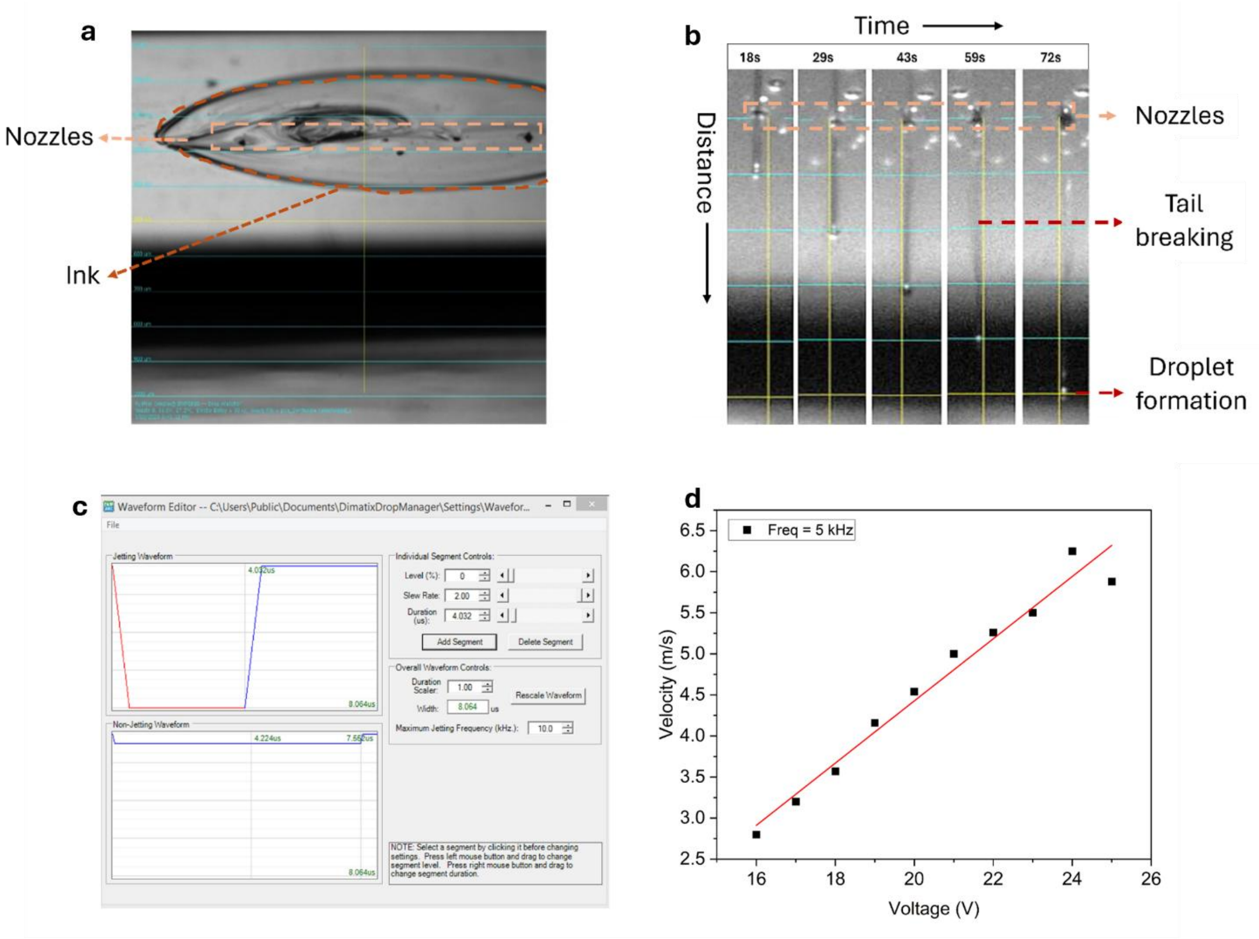

Figure S14. Drop watcher window view of (a) 4 wt% PVA (140 kDa) and (b) 4 wt% PVA (27 kDa) in a 2:1 water/DMSO mixture, captured using the drop watcher system of the Dimatix printer. (c) waveform used for the PVA-AuNTp ink, and (d) Dependence of the velocity of the droplet on the applied voltage of 27V.

Z number calculation

V_1 = 9.09, V_2 = 7.14, V_3 = 6.25, V_4 = 7.69. Average Velocity = 7.5 m/s.

Z number calculation for ink at 60°C.

Viscosity = 5.35 cP, Surface tension = 57.9 dyn/cm, Density 1.02 g/cm³.

L = 21 µm

$$Z = \frac{\sqrt{l\sigma\rho}}{\eta} = \frac{\sqrt{0.98\times\times 57.9\times 21}}{5.35} = 6.45$$

AAS (Atomic Absorption Spectroscopy) determination of Au concentration

Table 4: Summary of AAS results

Printed Mesh	Number of Layers	Concentration in 1 mL for a single mesh (µg/mL)
AuNP	10	1.87
	15	2.25
AuNTp	10	2
	15	3.25

Assessment of AuNTp Leaching and Aqueous Stability via UV-Vis Spectroscopy

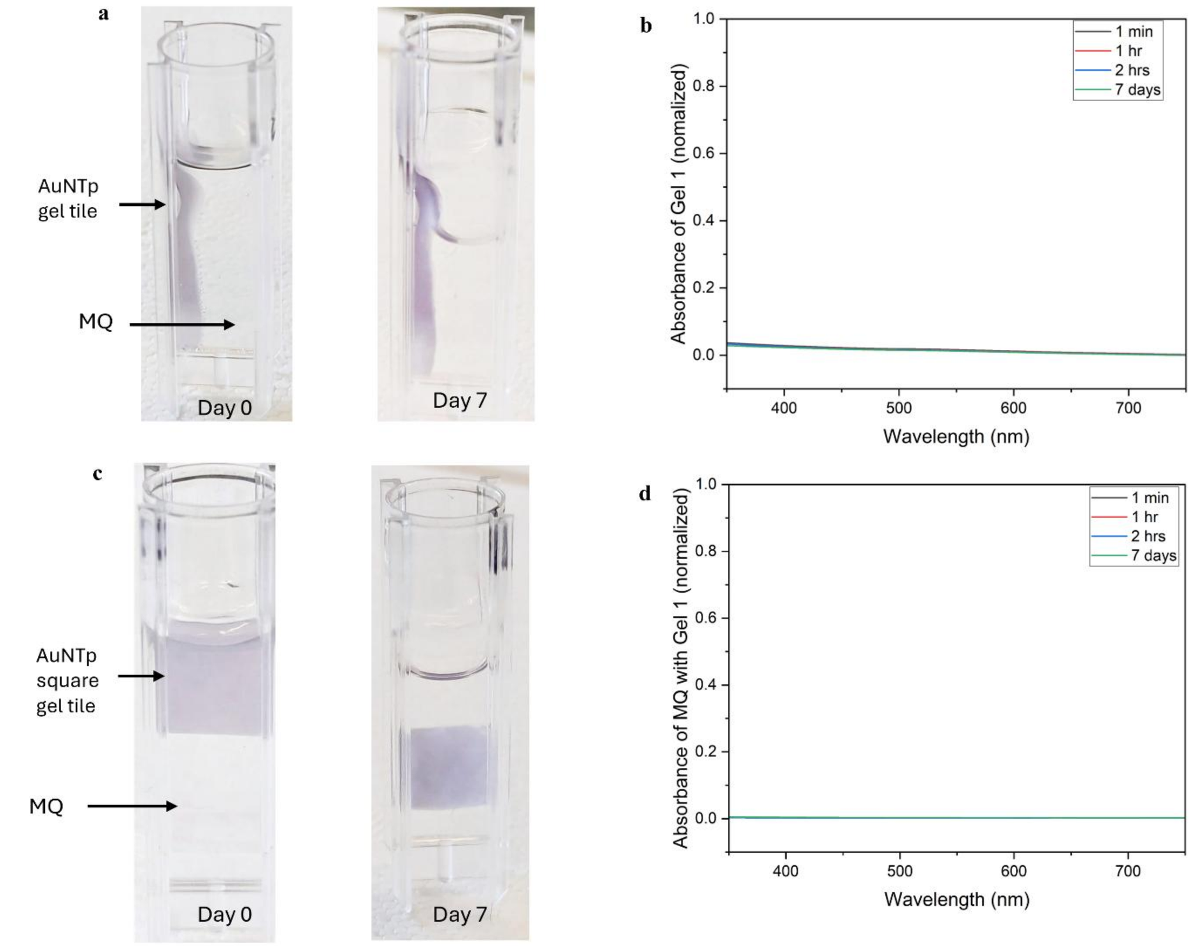

Figure S15 UV-Vis absorbance and visual assessment of AuNTp-PVA gel tiles in Milli-Q water over 7 days. (a, c) Photographs of AuNTp gel tiles at Day 0 and Day 7, showing physical integrity and swelling behaviour. (b) Normalised (0.1) absorbance spectra collected directly through the gel at 1 min, 1 h, 2 h, and 7 days, indicating minimal spectral variation and strong AuNTp retention. (d) Normalised (0.1) absorbance spectra of the surrounding Milli-Q water under leaching control conditions, showing no detectable AuNTp signal (500–700 nm) over the same time points, confirming negligible nanoparticle leaching.

Thickness measurement using Dektak

The thickness of the inkjet printed lines of different thicknesses was measured using a Dektak surface profilometer with a standard tip of 2 µm and a force of 3 mg before peeling off from the PET surface.

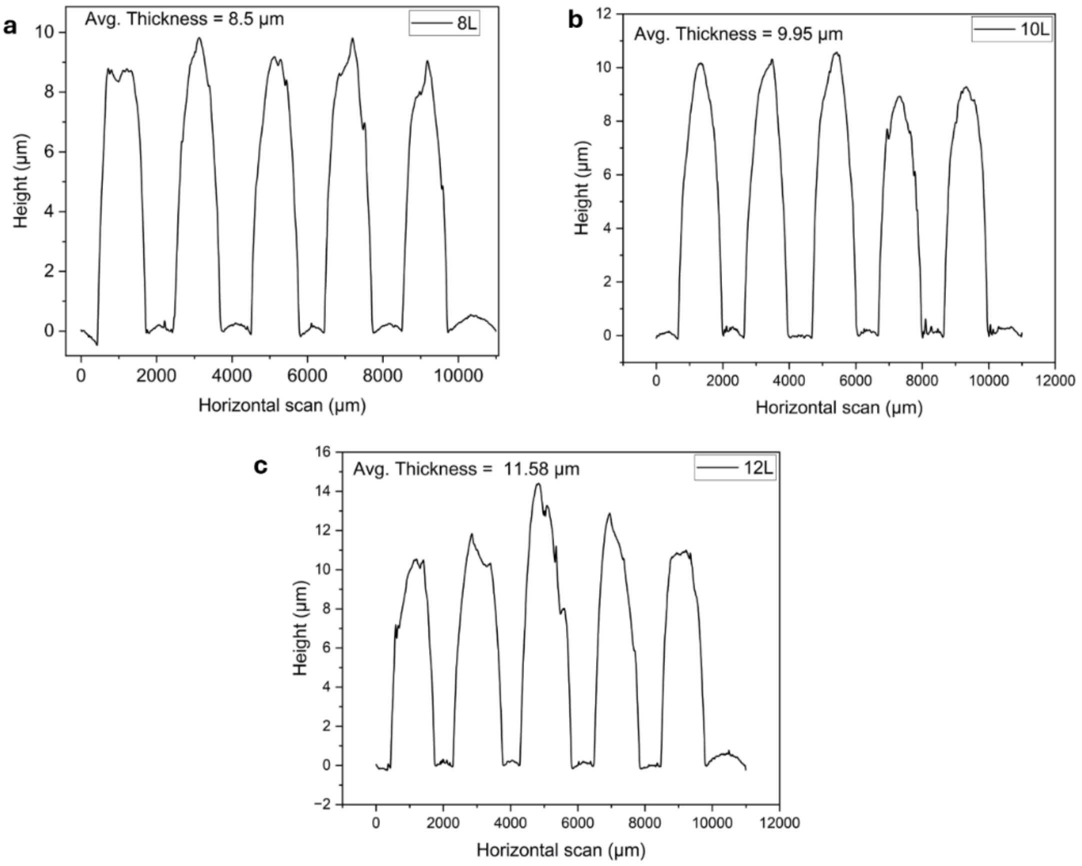

Figure S16. Thickness measurement of the inkjet printed lines using Dektak surface profilometer with (a) 8, (b) 10 and (c) 12 printed layers.

AuNTp/PVA-AuNP hydrogel meshes

The ink solution consists of 4 wt% PVA-AuNX with a water/DMSO ratio of (2/1). AuNX-PVA hydrogels were printed using a Dimatix materials printer 2850, operating in drop-on-demand mode with a piezoelectric printhead containing 16 nozzles (21 µL diameter, 10 pL drop volume). PET film of thickness 0.1mm purchased from Goodfellow, used as the printing substrate, pre-cleaned with Isopropyl alcohol and MQ water, and then dried with an N_2 gun. Printing parameters were optimised for droplet ejection and pattern formation: cartridge temperature at 60°C, platen temperature at 60°C for droplet spreading, a Dimatix model waveform was used, the meniscus level was adjusted to 5, and a 27V voltage was minimised to reduce splashing; the maximum jetting frequency was set at 5 kHz. Mesh structures were created by printing four horizontal lines (10 mm × 1 mm, 1 mm gap) first, then drying for 30 minutes, followed by four vertical lines of the exact dimensions. Pattern resolution was 1693 dpi, corresponding to 15 µm drop spacing. Print head angle adjusted to 3.4° based on drop spacing. After printing, the pattern dried for 30 minutes on the printer platen, which was heated at 60 ° C, then the PET film was peeled off.

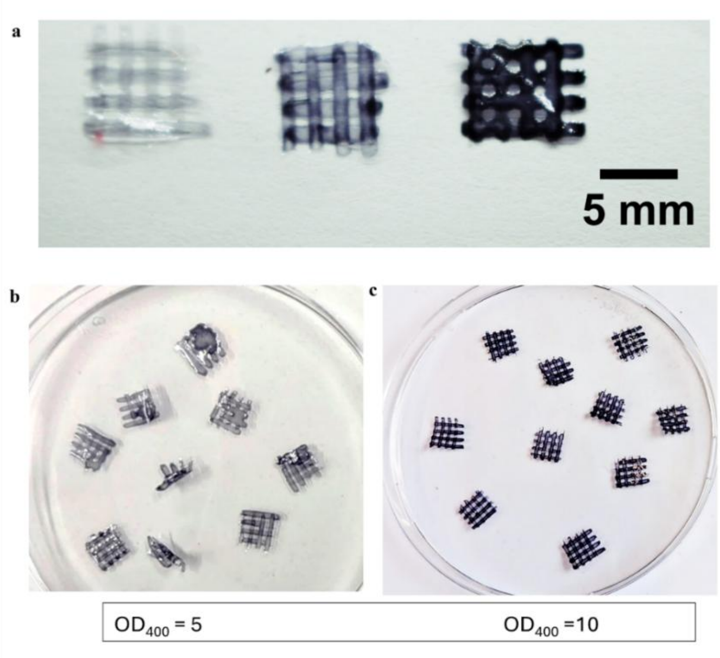

Figure S17 (a) Representative printed meshes using inks with OD_{400} = 2, 5, and 10, presented from left to right. ((b) Multiple meshes printed with OD_{400} = 5 ink, shown inside a petri dish. (c) Multiple meshes printed with OD_{400} = 10 ink, also shown in a petri dish. These images demonstrate the reproducibility and scalability of mesh formation at increasing nanoparticle loadings.

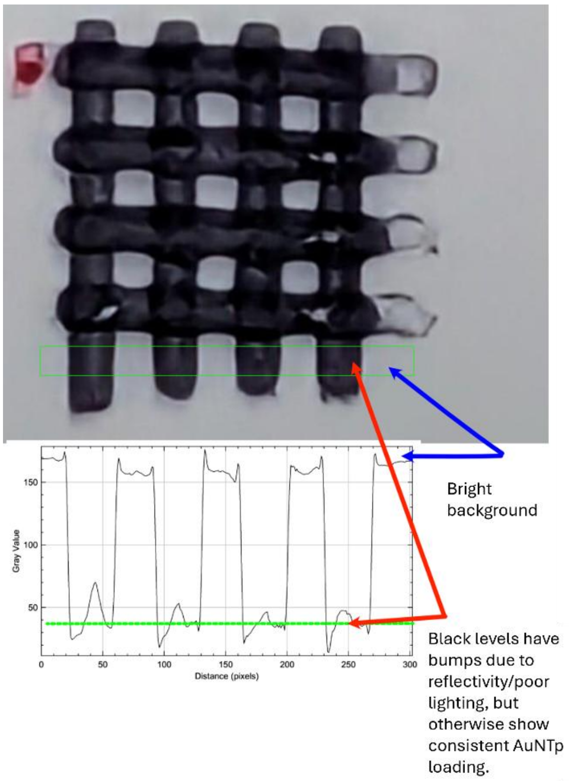

Figure S18 Digital image of an inkjet-printed PVA hydrogel mesh loaded with AuNTpps at OD_{400} = 10, alongside image analysis of grayscale intensity across the highlighted region (green box). The consistent peak profiles indicate uniform AuNTpp distribution within the printed strands. Minor variations in black levels are attributed to reflectivity and lighting conditions rather than uneven nanoparticle loading.

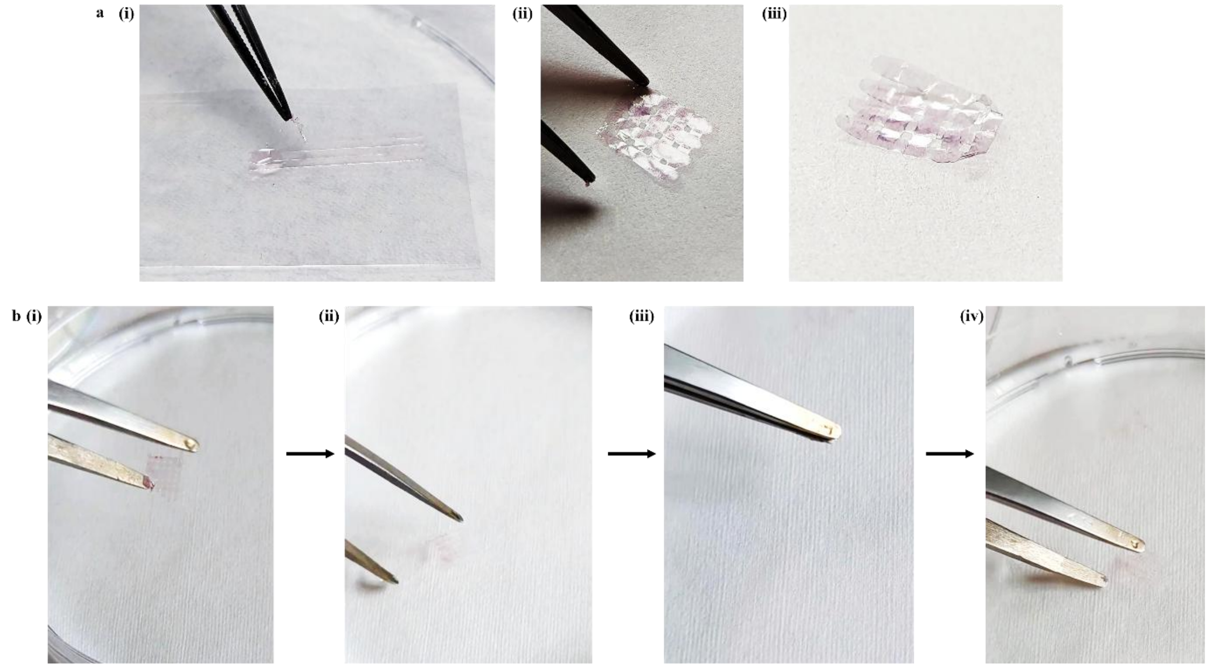

Figure S19 (a) Printing and collection of AuNP (OD_{400} = 2) thin meshes, showing shape-retaining and well-defined structures. (b) Handling tests of the meshes in aqueous medium: (i) intact mesh in petri dish, (ii–iv) lifting and transferring using tweezers, and (v–vi) demonstration of ease of manipulation without structural damage.

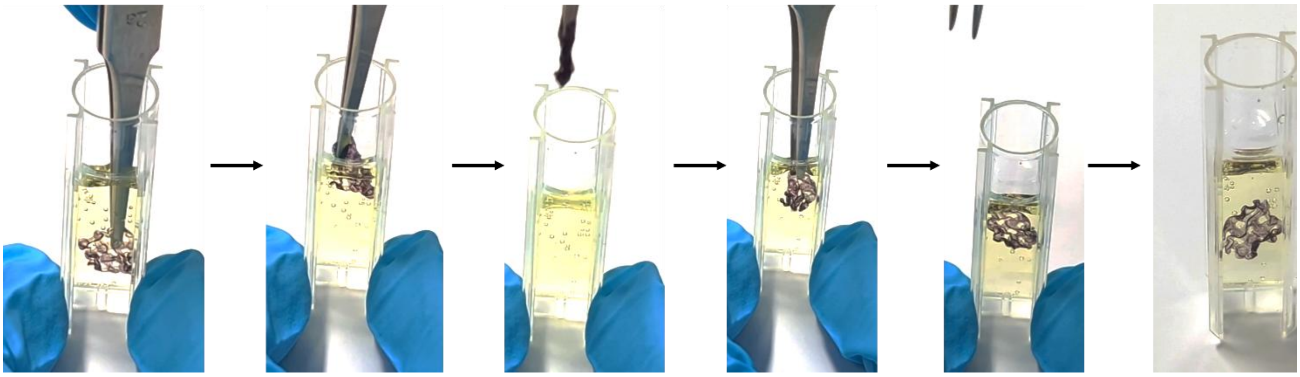

Figure S20 Sequential photographs illustrating the handling of AuNP meshes (OD_{400} = 10) in a 4-nitrophenol (4-NP) suspension. The meshes are (i) immersed in the reaction medium, (ii–iv) lifted out with tweezers, and (v–vi) reintroduced into the solution while maintaining their structural integrity and shape, demonstrating robustness and reusability in catalytic conditions.

Comparison of inkjet-printed meshes and gel tiles of AuNTp-PVA for 4-NP reduction

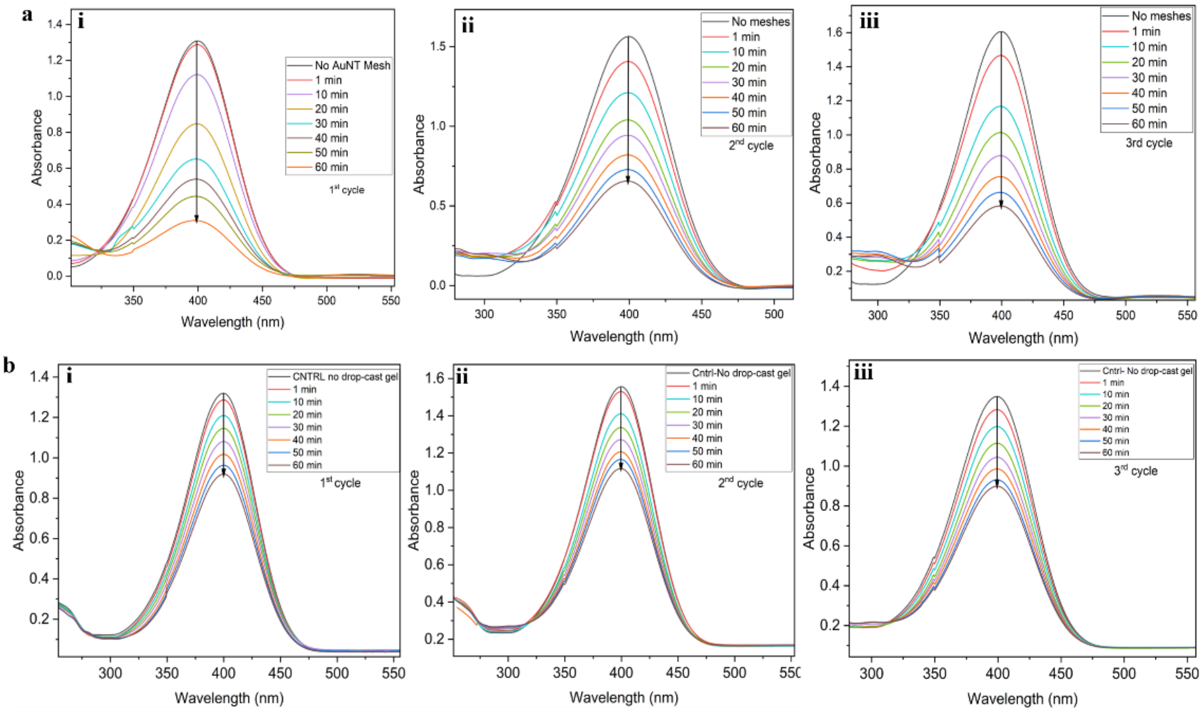

Figure S21. UV–Vis absorbance spectra showing the 4-NP reduction over three consecutive 60-minute cycles using 9 µg of AuNTp in (a) inkjet-printed meshes and (b) drop-cast gels. Each subpanel (i–iii) represents the 1st, 2nd, and 3rd cycle, respectively, compared with the corresponding control samples without AuNTp.

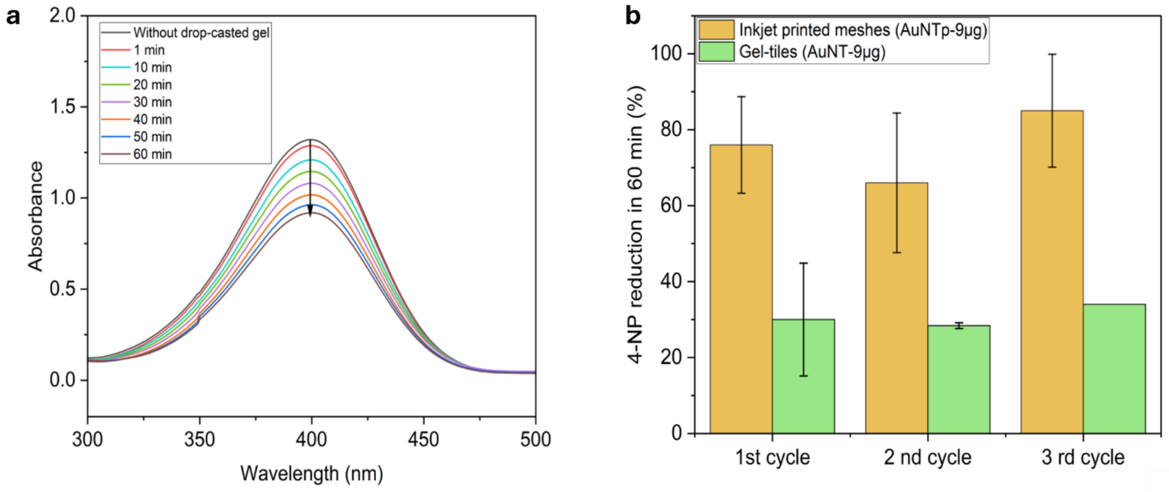

Figure S22 Evaluation of the Catalytic Performance of AuNTp in Inkjet-Printed Meshes and Gel Tiles for the Reduction of 4-Nitrophenol to 4-Aminophenol. (a) UV-Vis absorption spectra were recorded over 60 minutes using an AuNTp gel tile (9 µg), and (b) shows the comparison of activity for repeated cycles.

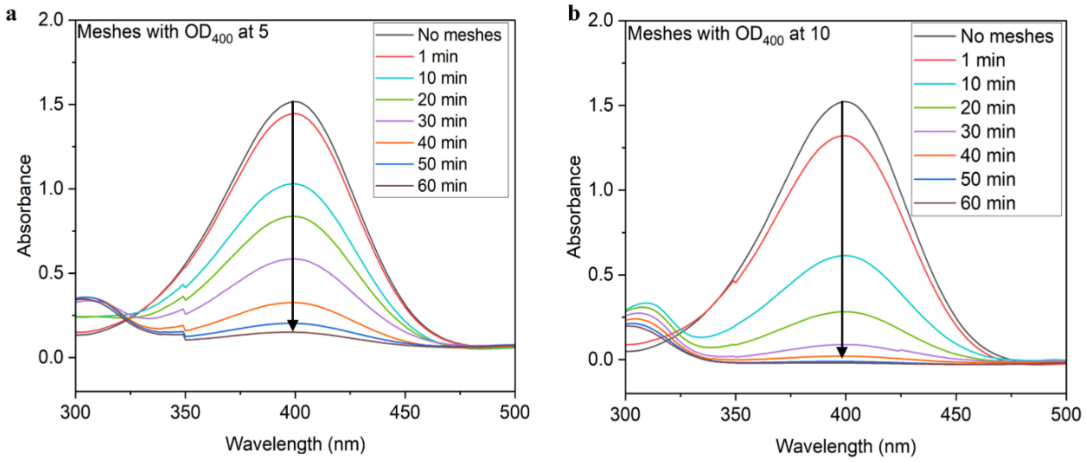

Figure S23. Comparison of Meshes Printed with Inks Containing Varying AuNTp Concentrations (OD_{400} at 5 and OD_{400} at 10) for the Reduction of 4-NP. UV-Vis absorption spectra were recorded at 10-minute intervals over 60 minutes with (a) OD_{400} at 5 and (b) OD_{400} at 10 meshes as catalysts. (c) Ln (C0/Ct) against time for k_{app} calculation. (d) Plot comparing the increase in AuNTp concentration in meshes and the apparent rate constant.

Oxidation of phenol using AuNTp-PVA meshes

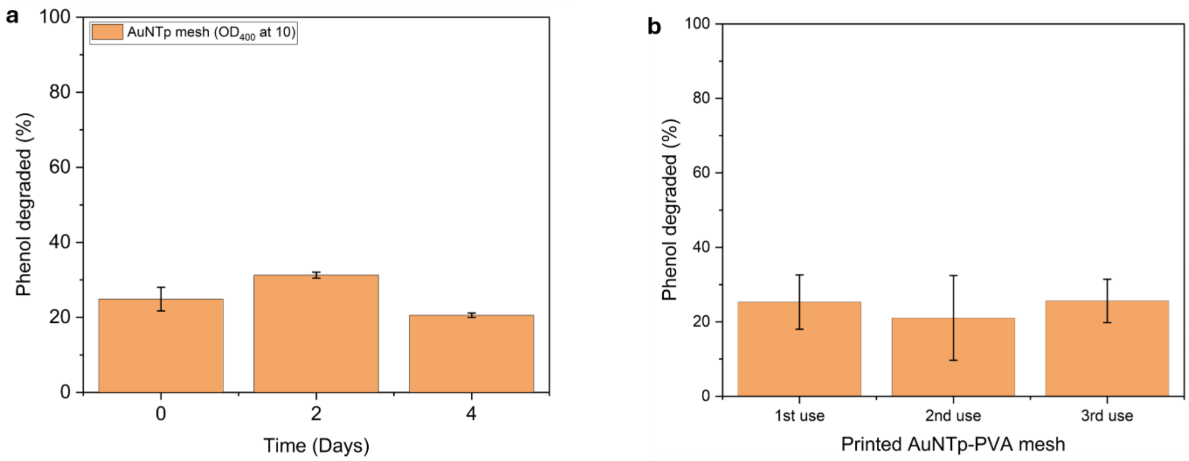

Figure S24 Inkjet-printed AuNTp meshes (OD_{400} = 10) were used for the oxidation of phenol with hydrogen peroxide at a phenol: H2O2 ratio of 1:2000. The reaction employed four meshes, each containing 90 µg of AuNTp, and was conducted at room temperature. (a) The percentage of phenol degraded over reaction periods of 0, 2, and 4 days was estimated using HPLC, and (b) demonstrates the reusability of the mesh over three consecutive reactions.

References

1 L. Vikingsson, A. Vinals-Guitart, A. Valera-Martínez, J. Riera, A. Vidaurre, G. Gallego Ferrer and J. L. Gómez Ribelles, *J Mater Sci*, 2016, **51**, 9979–9990.

2 L. Li and Y. Lo Hsieh, *Nanotechnology*, 2005, **16**, 2852–2860.

3 M. A. Monne, C. Q. Howlader, B. Mishra and M. Y. Chen, *Micromachines (Basel)*, 2021, **12**, 1–16.

4 I. Salaoru, Z. Zhou, P. Morris and G. J. Gibbons, *J Appl Polym Sci*, 2016, **133**, 1–9.

5 A. Babaie, S. Madadkhani and B. Stoeber, *Physics of Fluids*, DOI:10.1063/1.4868546.
